# ﻿Revision of the Chinese species of the genus *Brachyponera* Emery, 1900 (Hymenoptera, Formicidae), with a key to the world species of the genus

**DOI:** 10.3897/zookeys.1230.140159

**Published:** 2025-03-06

**Authors:** Chao Chen, Yu Yu, Chuanhui Yi

**Affiliations:** 1 Kunming Natural History Museum of Zoology, Kunming Institute of Zoology, Chinese Academy of Sciences, Kunming, Yunnan Province 650221, China; 2 Key Laboratory of Forest Disaster Warning and Control in Yunnan Province, College of Forestry, Southwest Forestry University, Kunming, Yunnan Province 650224, China; 3 Huanglianshan National Nature Reserve, Lvchun, Yunnan Province 662599, China; 4 Yunnan Institute of Biological Diversity, Southwest Forestry University, Kunming, Yunnan Province 650224, China

**Keywords:** High-resolution illustrations, new combination, new species, phylogenetics, Ponerinae, revision, taxonomy, Yunnan Province

## Abstract

The species of the ant genus *Brachyponera* Emery, 1900 are reviewed based on the morphological characters of the worker caste. *Brachyponeratianzun* (Terayama, 2009) is transferred to *Euponera* as *Euponeratianzun* (Terayama, 2009), **comb. nov.** Four new species, *B.paraarcuata***sp. nov.**, *B.xui***sp. nov.**, *B.candida***sp. nov.**, and *B.myops***sp. nov.**, are described from China based on the worker caste. Phylogenetic trees were constructed for eight known and four new species, and genetic distances were calculated. High-resolution images of *B.batak* (Yamane, 2007), *B.flavipes* (Yamane, 2007), *B.nakasujii* (Yashiro et al., 2010), *B.pilidorsalis* (Yamane, 2007), *B.wallacea* (Yamane, 2007), and *B.brevidorsa* Xu, 1994 are provided. Keys are provided to both the eight species groups and 23 known species of the world based on the worker caste.

## ﻿Introduction

The genus *Brachyponera* Emery, 1900, was initially established as a subgenus of *Euponera* Forel, 1891 with the type species E. (B.) croceicornis (Emery, 1900), and later elevated to the genus level (Bingham 1903). Subsequently, it was synonymized with the genus *Pachycondyla* F. Smith, 1858 (Snelling 1981) and placed within the tribe Ponerini (Bolton 2003). Since the 21^st^ century, with the widespread application of molecular phylogenetic analysis, the subgenus Brachyponera has been reinstated as an independent genus and placed under the *Odontomachus* genus group of the tribe Ponerini (Schmidt 2013; Schmidt and Shattuck 2014).

*Brachyponera* workers have a small body size and are carnivorous or scavenging, typically choosing to construct their nests in decaying wood or soil (Wheeler 1933; Haskins and Haskins 1950; Wilson 1958; Ogata 1987; Matsuura 2002; Matsuura et al. 2002; Kikuchi et al. 2007; Yamane 2007; Gotoh and Ito 2008). Two species of the genus known from Thailand are cave dwelling ants (*B.kumtongi* and *B.troglomorpha*). Ant crickets (*Myrmecophilus* Berthold, 1827) were found together with foraging workers of the species (Duanchay et al. 2024). *Brachyponera* is unusual among ponerines in that it displays a marked reproductive dimorphism between workers and queens, with the workers having completely lost their reproductive organs and queens having a large number of ovarioles (Ito and Ohkawara 1994; Gotoh and Ito 2008).

*Brachyponera* is widespread from Africa through southern Asia to Australia. It is the most species-rich in Southeast Asia (Duanchay et al. 2024). *Brachyponerachinensis* (Emery) was accidentally introduced to the southeastern United States and is now locally abundant (Nelder et al. 2006); it has also been introduced to New Zealand and Europe (Schmidt and Shattuck 2014; Menchetti et al. 2022; Duanchay et al. 2024). Currently, 20 valid species and five valid subspecies are recognized in the world (Bolton 2024). In this work, we focused only on the species level taxa due to their clear-cut criteria and data availability, while subspecies classification, which is more complex and variable, could introduce ambiguity and complicate our analysis.

*Brachyponerasennaarensis* (Mayr, 1862) is widely distributed in central to southern regions of Africa, representing the single species in Africa. The genus is represented by one species in Korea and Laos, two species in Australia and New Guinea, three species in Java, Sulawesi, Philippines, Sumatra, Malaysia, Japan, Myanmar, and Sri Lanka, four species in Vietnam and India, and five species in Thailand (Janicki et al. 2016). In China, five species have been recorded (Guénard et al. 2017). Currently, *B.brevidorsa* Xu, a species endemic to China, represents the only species within the genus described by a Chinese researcher (Xu 1994). The other taxon described as a member of *Brachyponera* was later transferred to the genus *Hypoponera* and is currently known as *H.mesoponeroides* (Dang et al. 2018).

It appears that the intergeneric transfers within Ponerini are still unresolved cases, as the results of our work revealed another species with a wrongly assigned genus. Terayama (2009) described *Pachycondylatianzun* based on workers collected from Taiwan, China. Later, in 2014, Schmidt and Shattuck reinstated *Brachyponera* as a separate genus and reclassified *Pachycondylatianzun* as a member of *Brachyponera*. Examining the original description of the species with photographs of the type specimen in the Institute of Agro-Environmental Research, Japan (https://www.naro.affrc.go.jp/archive/niaes/inventory/insect/inssys/hymlst.htm HYM-182), we have concluded the characterization of this species does not correspond with *Brachyponera*, but well agrees with *Euponera* in some important diagnostic characters separating the two genera. Therefore, in the present paper, *B.tianzun* is transferred to *Euponera* as a new combination. The species is redescribed below on the basis of the original description and photographs of the holotype specimen.

In this study, we review the Chinese species of *Brachyponera*, with descriptions of four new species from Yunnan, China, accompanied by high-resolution images and measurements of important morphological characters. We also provide a synoptic list of 23 extant species of the genus and a key to their determination. A key to species groups based on the worker caste is also provided.

## ﻿Materials and methods

The ant specimens were obtained using sample-plots and search-collecting methods (e.g., Xu 2002). Sample plots were set up at the research site with different altitude gradients and included a variety of vegetation types within the survey area. Subsequently, the specimens were meticulously examined using an SDPTOP-SZM stereomicroscope. High-quality multifocus montage images were captured using a Keyence VHX-6000 ultra-depth microscopic three-dimensional microscope. To compare the worker morphology of the four new species, reference was made to the original descriptions of related species (Mayr 1862; Emery 1895; Karavaiev 1925). Sculptural and hair terminology follows Harris (1979) and Wilson (1955). The key was prepared using the examined specimens, images available on AntWeb (2024) and AntWiki (2024), and original descriptions of the species.

The investigated material is deposited in the following institutions:

**KIZ**Kunming Natural History Museum of Zoology, Kunming Institute of Zoology, Chinese Academy of Sciences, Kunming, Yunnan Province, China.

**SWFU** Insect Collection, Southwest Forestry University, Kunming, Yunnan Province, China.

**GXNU**Insect Collection, Guangxi Normal University, Guilin, Guangxi, China.

Standard measurements and indices were employed as defined in Bolton (1975) and Lattke (2011), with the addition of mandible length and eye diameter as outlined below. Furthermore, alitrunk (mesosoma) length is substituted by Weber’s Length in accordance with the methodology proposed by Xu and He (2015). Head lateral margin length, anterior head length, clypeal median lobe length, pronotum length and pronotum height were also measured (see Arimoto 2017). All measurements are expressed in millimeters.

**HL** Head length: the straight-line length of the head in perfect full-face view, measured from the midpoint of the anterior clypeal margin to the midpoint of the posterior margin. In species where one or both of these margins are concave, the measurement is taken from the mid-point of a transverse line that spans the apices of the projecting portions.

**HLL** Head lateral margin length: in full-face view, the head length measured from the mandible base to the nuchal carina.

**HLA** Anterior head length: in full-face view, the head length measured from the mandible base to the anterior edge of the eye.

**HW** Head width: the maximum width of the head in full-face view, excluding the eyes.

**ML** Mandible length: the straight-line length of the mandible measured from the apex to the lateral base.

**CML** Clypeal median lobe length: in full-face view, the straight-line length measured from the anterior margin of the clypeus to the anterior margin of the torulus.

**SL** Scape length: the straight-line length of the antennal scape, excluding the basal constriction or neck.

**ED** Eye diameter: the maximum diameter of the eye.

**PrL** Pronotum length: in profile, the diagonal length of the pronotum, measured from the anterior margin of the pronotum excluding the collar to the posterior extremity of the pronotum.

**PrH** Pronotum height: in profile, the maximum height of the pronotum, measured from the posterior base of the lateral margin of the pronotum to the highest point of the pronotum.

**PrW** Pronotum width: the maximum width of the pronotum measured in dorsal view.

**WL** Weber’s length (= alitrunk length): the diagonal length of the mesosoma in lateral view, measured from the point at which the pronotum meets the cervical shield to the posterior basal angle of the metapleuron.

**TL** Total length: the total outstretched length of the individual, from the mandibular apex to the gastral apex.

**PL** Petiole length: the length of the petiole measured in lateral view from the anterior process to the posteriormost point of the tergite, where it surrounds the gastral articulation.

**PH** Petiole height: the height of the petiole measured in lateral view from the apex of the ventral (subpetiolar) process vertically to a line intersecting the dorsal most point of the node.

**DPW** Dorsal petiole width: the maximum width of the petiole in dorsal view.

**CI** Cephalic Index = HW × 100 / HL.

**SI** Scape index = SL × 100 / HW.

**LPI** Lateral petiole index = PH × 100 / PL.

**PDPI** Dorsal petiole index = DPW × 100 / PL.

**Other abbreviations. w.**: worker; **q.**: queen; **m.**: male; **var.**: variety; **subsp.**: subspecies.

DNA extraction of tissue fragments from ants was made using TSINGKE TSP202-50 Trelief® Hi-Pure Animal Genomic DNA Kit. The standard COI barcoding fragment (Hebert et al. 2003) was amplified using a cocktail of primers LCO1490 (GGTCAACAAATCATAAAGATATTGG) and HCO2198 (TAAACTTCAGGGTGACCAAAAAATCA) (Folmer et al. 1994). Amplification was performed with synthesized primers using TSINGKE Gold Mix (green), PCR reactions contained 47ul Gold Mix (green), 1 ul 10 µM Primer F, 1 ul 10 µM Primer R, with 1.0 µl of template DNA. PCR was performed using an initial denaturation step of 2 min at 98 °C, followed by 30 cycles of 10 s at 98 °C, 10 s at 50 °C and 10 s at 72 °C, and finishing with an extension of 5 min at 72 °C and pause at 4 °C. The amplified PCR products were subjected to agarose gel electrophoresis (2 µl sample + 6 µl bromophenol blue) at 300V for 12 min to obtain the identification gel graphs. The products were purified and sequenced by Tsingke Biotechnology (Beijing) Co., Ltd., using the same primers as in PCR. Sequences were edited and manually managed using SeqMan in Lasergene v. 7.1 (DNASTAR Inc., Madison, WI, USA) and MEGA 11 (Tamura et al. 2021). All new sequences were deposited in GenBank. *Hypoponeramesoponeroides* (Radchenko) and *Euponerasikorae* (Forel) were chosen as outgroups, and their sequences along with seven described *Brachyponera* species were obtained from GenBank (Table 1).

**Table 1.** Sequences used in this study.

Species	GenBank	Voucher/isolate	Length	AT%
*Brachyponerabrevidorsa*	> PQ863325	KIZ20231327	681	72.6
*Brachyponeracandida* sp. nov.	> PQ863326	KIZ20230168	677	70.9
*Brachyponerachinensis*	> OM604749	MM21B056a1	658	71.6
*Brachyponeracroceicornis*	> PP069658	HP0159	659	69.8
*Brachyponeraluteipes*	> LC426718	Nago5	565	68
*Brachyponeramyops* sp. nov.	> PQ863327	KIZ20231049	676	72.2
*Brachyponeranakasujii*	> MH370729	Bnak4	543	68.2
*Brachyponeranigrita*	> GQ264596	63	565	71
*Brachyponeraobscurans*	> EF609940	> CASENT0060572-D01	657	70
*Brachyponeraparaarcuata* sp. nov.	> PQ863328	KIZ20231657	680	69.8
*Brachyponerasennaarensis*	> OR527449	RyadhLM2019	645	71.8
*Brachyponeraxui* sp. nov.	> PQ863329	KIZ20231023	679	72
*Euponerasikorae*	> HQ925507	> CASENT0152189-D01	635	70.1
*Hypoponeramesoponeroides*	> LC349924	AD170525-04	558	74

Sequences were aligned using ClustalW (Thompson et al. 2002) with default parameters in MEGA 11 (Tamura et al. 2021) with default parameters. The genetic divergence (uncorrected p-distance) between species was calculated in MEGA 11 (Tamura et al. 2021). The best substitution model GTR+G+I was selected using the Akaike Information Criterion (AIC) in ModelFinder (Nei and Kumar 2000).

Evolutionary history was inferred by using the maximum likelihood method and the General Time Reversible model (Nei and Kumar 2000). The tree with the highest log likelihood (-3345.33) is shown. The percentage of trees in which the associated taxa clustered together is shown below the branches. Initial tree(s) for the heuristic search were obtained automatically by applying neighbor-joining and BioNJ algorithms to a matrix of pairwise distances estimated using the Maximum Composite Likelihood (MCL) approach and then selecting the topology with superior log likelihood value. A discrete Gamma distribution was used to model evolutionary rate differences among sites (5 categories (+G, parameter = 0.8794)). The rate variation model allowed for some sites to be evolutionarily invariable ([+I], 50.92% sites). The tree is drawn to scale, with branch lengths measured in the number of substitutions per site. This analysis involved 14 nucleotide sequences. There was a total of 668 positions in the final dataset. Evolutionary analyses were conducted in MEGA11 (Tamura et al. 2021), and nodal support was estimated by 1000 rapid bootstrap replicates. Bayesian inference phylogenies were inferred using MrBayes v. 3.2.7a (Ronquist et al. 2012) under the JC model (2 parallel runs, 2000000 generations), in which the initial 25% of sampled data were discarded as burn-in. Phylogenetic trees were edited with iTOL v. 5 (Letunic and Bork 2021).

## ﻿Results

**Figure 1.**
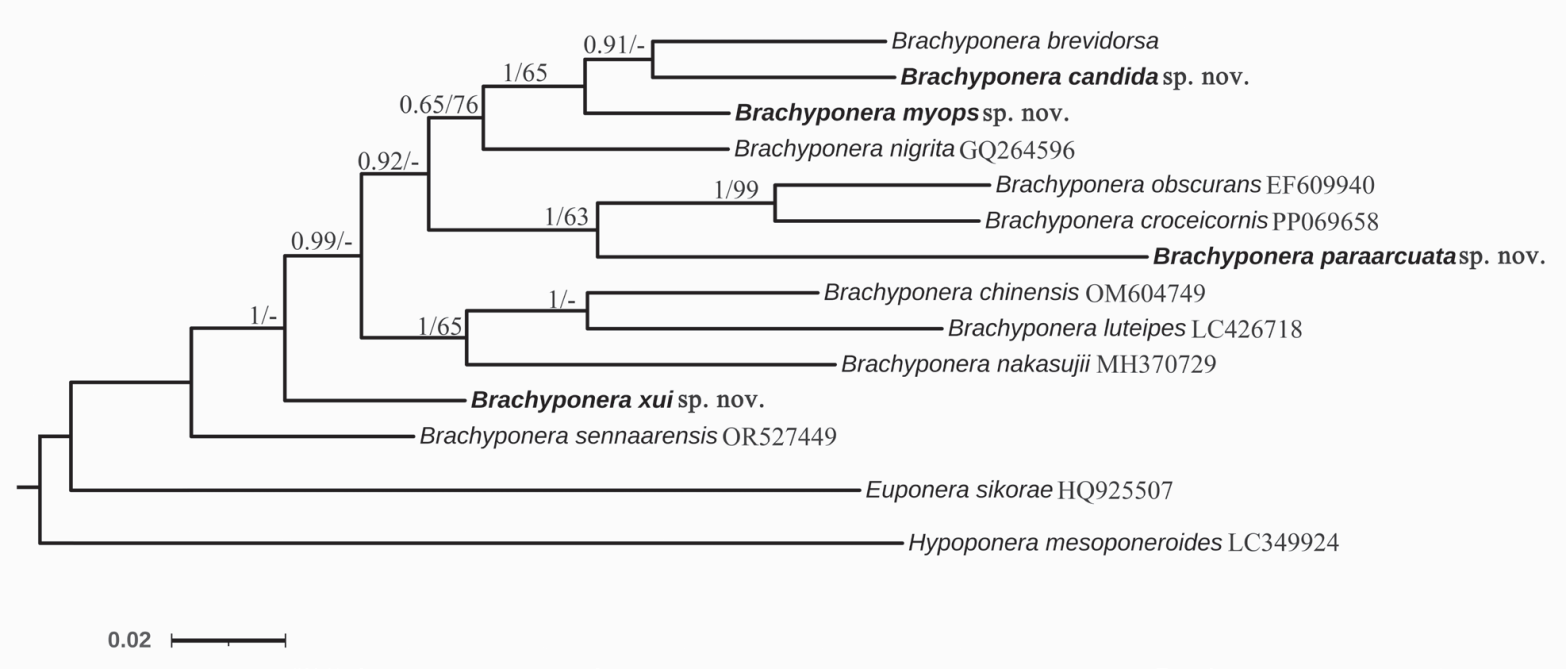
Phylogenetic tree inferred from Bayesian analysis based on the COI gene. Numbers before slashes indicate Bayesian posterior probabilities (only values above 0.6 are shown) and numbers after slashes indicate bootstrap support from maximum likelihood analysis (only values above 60 are shown).

We obtained COI gene sequences of seven species of *Brachyponera* and two outgroups from GenBank (Table 1). We determined COI gene sequences de novo for the four new species and *B.brevidorsa* Xu, resulting in a total of 14 nucleotide sequences. The generated gene sequences were 668 bp in length. The topologies derived from Bayesian inference and maximum likelihood analyses were consistent (Fig. 1).

The 12 species of the genus *Brachyponera* were all grouped together and distinguished from the two outgroup species. *Brachyponerasennaarensis* and *B.xui* sp. nov. were located at the base of the tree. The genetic distance between *B.candida* sp. nov., *B.myops* sp. nov. and *B.brevidorsa* was relatively close, resulting in the three species grouping together. *B.paraarcuata* sp. nov. is a relatively derived species; the genetic distance between it and other species is greater than 0.13, and it is clustered with *B.obscurans* and *B.croceicornis*. *B.chinensis*, *B.luteipes* and *B.nakasujii* are grouped together (Table 2).

**Table 2.** Estimates of evolutionary divergence between sequences.

		1	2	3	4	5	6	7	8	9	10	11	12	13
1	*B.chinensis*													
2	*B.croceicornis*	0.1398												
3	*B.luteipes*	0.0980	0.1385											
4	*B.nakasujii*	0.1154	0.1471	0.1258										
5	*B.nigrita*	0.1184	0.1487	0.1263	0.1194									
6	*B.obscurans*	0.1370	0.0700	0.1408	0.1517	0.1367								
7	*B.sennaarensis*	0.1226	0.1274	0.1389	0.1480	0.1197	0.1370							
8	*H.mesoponeroides*	0.1935	0.1989	0.1868	0.2182	0.1936	0.2043	0.1756						
9	*E.sikorae*	0.1874	0.1937	0.2163	0.2158	0.1939	0.2142	0.1680	0.2204					
10	*brevidorsa*	0.1079	0.1290	0.1303	0.1343	0.0957	0.1172	0.1163	0.1900	0.1827				
11	*B.candida* sp. nov.	0.1125	0.1214	0.1283	0.1407	0.0978	0.1157	0.1302	0.1989	0.1843	0.0793			
12	*B.myops* sp. nov.	0.1049	0.1184	0.1242	0.1173	0.0754	0.1111	0.1023	0.1935	0.1732	0.0704	0.0704		
13	*B.paraarcuata* sp. nov.	0.1565	0.1366	0.1670	0.1962	0.1507	0.1492	0.1519	0.2061	0.2000	0.1497	0.1482	0.1437	
14	*B.xui* sp. nov.	0.1003	0.1123	0.1059	0.1130	0.0896	0.1111	0.0946	0.1738	0.1843	0.1123	0.1123	0.0868	0.1452

Note: the number of base differences per site from between sequences is shown. Values represent the genetic distance between two different species. This analysis involved 14 nucleotide sequences. All ambiguous positions were removed for each sequence pair (pairwise deletion option). There was a total of 668 positions in the final dataset. Evolutionary analyses were conducted in MEGA11 (Tamura et al. 2021).

### ﻿Key to species groups of *Brachyponera* based on workers

**Table:** 

1	Lateral face of pronotum with transverse rugae	***atrata* group**
–	Lateral face of pronotum smooth and shiny or only punctate	**2**
2	In full-face view, head broader than long	***lutea* group (part)**
–	In full-face view, head longer than broad	**3**
3	In full-face view, eye small, with five ommatidia along longest axis (ED 0.07 mm) (China)	***kumtongi* group**
–	In full-face view, eye moderately large, with more than nine ommatidia along longest axis (ED > 0.15 mm)	**4**
4	In lateral view, propodeum densely transversely rugose; smooth area if any much restricted	***chinensis* group**
–	In lateral view, propodeum smooth or only punctate	**5**
5	In dorsal view, lateral margin of pronotum with well-defined edge; lateral slope of pronotum nearly vertical	***xui* group**
–	In dorsal view, lateral margin of pronotum not well defined; lateral slope of pronotum convex	**6**
6	In lateral view, dorsal surface of propodeum continuously connected with declivity, forming complete circular arc	**7**
–	In lateral view, dorsal surface of propodeum clearly differentiated from declivity; posterodorsal corner of propodeum roundly angled	**8**
7	Larger species, > 5 mm in total body length; antenna scape long, > 1/5 exceeds posterolateral corner; in lateral view, pronotum weakly convex	***arcuata* group**
–	Smaller species, < 4 mm in total body length; antenna scape short, slightly exceeds posterolateral corner; in lateral view, pronotum relatively flat	***lutea* group (part)**
8	Larger species, > 4.5 mm in total body length; head width > 0.9 mm; flagellar segments 1 and 2 longer than broad; body color usually dark brown	***nigrita* group**
–	Smaller species, < 4.5 mm in total body length; head width < 0.9 mm; flagellar segments 1 and 2 each as long as broad, or broader than long; body color usually yellowish brown to tan	***obscurans* group**

### ﻿Synoptic list of *Brachyponera*

#### ﻿*Brachyponeraatrata* group

***Brachyponeraatrata* (Karavaiev, 1925)** (Suppl. material 1: fig. S1)

Euponera (Brachyponera) atrata Karavaiev, 1925b: 126 (w.) Indonesia (Ambon I.).

***Brachyponerawallacea* (Yamane, 2007)** (Suppl. material 1: fig. S2)

*Pachycondylawallacea* Yamane, 2007: 659, fig. 8 (w.q.) Indonesia (Lombok I.).

*Brachyponerawallacei* [sic]: Schmidt and Shattuck 2014: 81.

#### ﻿*Brachyponeralutea* group

***Brachyponeralutea* (Mayr, 1862)** (Suppl. material 1: fig. S3)

*Poneralutea* Mayr, 1862: 721 (w.q.) Australia (New South Wales).

***Brachyponerasennaarensis* (Mayr, 1862)** (Suppl. material 1: figs S4, S5)

*Ponerasennaarensis* Mayr, 1862: 721 (w.) Sudan.

***Brachyponerabrevidorsa* Xu, 1994** (Fig. 22)

*Brachyponerabrevidorsa* Xu, 1994b: 183, figs 5, 6 (w.) China (Yunnan).

*Brachyponeratroglomorpha* Duanchay & Jaitrong, 2024

*Brachyponeratroglomorpha* Duanchay & Jaitrong, in Duanchay et al. 2024: 7, figs 2, 3C, 4 (w.) Thailand. Indomalaya.

#### ﻿*Brachyponerakumtongi* group

***Brachyponerakumtongi* Duanchay & Jaitrong, 2024**

*Brachyponerakumtongi* Duanchay & Jaitrong, in Duanchay et al. 2024: 4, figs 1, 3B, 4 (w.) Thailand. Indomalaya.

***Brachyponeramyops* Chen, Yu & Yi, sp. nov.** (Fig. 26). China (Yunnan).

*Brachyponerachinensis* group

***Brachyponerachinensis* (Emery, 1895)** (Fig. 24)

Poneranigritasubsp.chinensis Emery, 1895 m: 460 (w.) China (Shanghai). Palearctic.

***Brachyponeranakasujii* (Yashiro et al., 2010)** (Suppl. material 1: fig. S6)

*Pachycondylanakasujii*Yashiro et al., 2010: 44, figs 4, 5 (w.q.m.) Japan.

#### ﻿*Brachyponeraxui* group

***Brachyponeraxui* Chen, Yu & Yi, sp. nov.** (Fig. 30). China (Yunnan).

#### ﻿*Brachyponeraarcuata* group

***Brachyponeraarcuata* (Karavaiev, 1925)** (Suppl. material 1: figs S7, S8)

Euponera (Brachyponera) luteipes var. arcuata Karavaiev, 1925b: 125, figs 3c, 4 (w.q.m.) Indonesia (Java).

***Brachyponeraparaarcuata* Chen, Yu & Yi, sp. nov.** (Fig. 29). China (Yunnan).

#### ﻿*Brachyponeranigrita* group

***Brachyponerabatak* (Yamane, 2007)** (Suppl. material 1: fig. S9)

*Pachycondylabatak* Yamane, 2007: 656, figs 3, 4, 12–16 (w.q.m.) Indonesia (Sumatra).

***Brachyponeracandida* Chen, Yu & Yi, sp. nov.** (Fig. 23). China (Yunnan).

***Brachyponeraflavipes* (Yamane, 2007)** (Suppl. material 1: fig. S10)

*Pachycondylaflavipes* Yamane, 2007: 658, fig. 5 (w.) Myanmar.

***Brachyponeranigrita* (Emery, 1895)** (Fig. 27)

*Poneranigrita* Emery, 1895 m: 459 (w.) Myanmar.

***Brachyponerapilidorsalis* (Yamane, 2007)** (Suppl. material 1: fig. S11)

*Pachycondylapilidorsalis* Yamane, 2007: 655, figs 7, 9–11 (w.q.) West Malasia.

#### ﻿*Brachyponeraobscurans* group

***Brachyponerachristmasi* (Donisthorpe, 1935)** (Suppl. material 1: fig. S12)

Euponera (Mesoponera) christmasi Donisthorpe, 1935: 630 (w.) Christmas Island.

***Brachyponeracroceicornis* (Emery, 1900)** (Suppl. material 1: fig. S13)

Euponera (Brachyponera) luteipes var. croceicornis Emery, 1900b: 315 (w.q.) New Guinea (Papua New Guinea).

***Brachyponerajerdonii* (Forel, 1900)** (Suppl. material 1: fig. S14)

*Ponerajerdonii* Forel, 1900f: 327 (w.) India (Maharashtra, West Bengal, Kerala, Assam).

***Brachyponeraluteipes* (Mayr, 1862)** (Fig. 25)

*Poneraluteipes* Mayr, 1862: 722 (w.q.) India (Nicobar Is).

***Brachyponeraobscurans* (Walker, 1859)** (Fig. 28)

*Formicaobscurans* Walker, 1859: 372 (q.) Sri Lanka.

### ﻿Key to *Brachyponera* species based on the worker caste

The distinction between the four species, *B.flavipes*, *B.batak*, *B.pilidorsalis*, and *B.nigrita* is principally based on Yamane (2007).

**Table:** 

1	Lateral face of pronotum with transverse rugae (Fig. 2A, B)	**2**
–	Lateral face of pronotum smooth and shiny or only punctate (Fig. 2C, D)	**3**
2	Dorsum of propodeal declivity with rugae only on upper part (Fig. 3A); in lateral view, propodeum with sparsely transverse rugae (Fig. 3C) (Maluku islands)	***B.atrata* (Karavaiev)**
–	Dorsum of propodeal declivity with rugae on middle and upper parts (Fig. 3B); in lateral view, propodeum with densely transverse rugae (Fig. 3D) (Sulawesi, Lombok, and Bali)	***B.wallacea* (Yamane)**
3	Eye small with 2–5 ommatidia along longest axis (ED 0.07 mm) (Fig. 4A, B)	**4**
–	Eye moderately large, with > 7 ommatidia along longest axis (ED > 0.10 mm) (Fig. 4C, D)	**5**
4	Eye very small, present as a dot, with 2 or 3 ommatidia along longest axis (Fig. 4B); antennal segments 3 and 4 each as long as broad, or broader than long; body color reddish brown (Thailand)	***B.kumtongi* Duanchay & Jaitrong**
–	Eye relatively small, with 4 or 5 ommatidia along longest axis (Fig. 4A); antennal segments 3 and 4 each longer than broad; body color brownish black (China)	***B.myops* Chen, Yu & Yi, sp. nov.**
5	In full-face view, head broader than long, or almost as long as broad (Fig. 5A, B)	**6**
–	In full-face view, head longer than broad (Fig. 5C, D)	**7**
6	Posterior margin of head in in full-face view strongly concave; scape long, slightly extending beyond posterolateral corner of head; eye large (Fig. 5A); body color dark brown (Fig. 6A) (Central to Southern Africa)	***B.sennaarensis* (Mayr)**
–	Posterior margin of head in full-face view weakly concave; scape short, not reaching posterolateral corner of head; eye small (Fig. 5B); body color yellow brown (Fig. 6B) (Australia)	***B.lutea* (Mayr)**
7	In lateral view, propodeum densely transversely or irregularly rugose (Fig. 7A, B)	**8**
–	In lateral view, propodeum smooth or only punctate (Fig. 7C, D)	**9**
8	In dorsal view, petiolar node broader (PDPI 175) (Fig. 8A, B) (Japan)	***B.nakasujii* (Yashiro et al.)**
–	In dorsal view, petiolar node narrow (PDPI 130) (Fig. 8C, D) (China, North Korea, South Korea, Japan, Vietnam, Thailand, Nepal)	***B.chinensis* (Emery)**
9	In dorsal view, lateral margin of pronotum markedly edged; lateral face of pronotum nearly vertical (Fig. 9A) (China)	***B.xui* Chen, Yu & Yi, sp. nov.**
–	In dorsal view, lateral margin of pronotum without well-defined edges; lateral faces of pronotum diverging downward, appearing more convex (Fig. 9B)	**10**
10	In lateral view, dorsal surface of propodeum continuously connected with declivity, forming complete circular arc (Fig. 10A)	**11**
–	In lateral view, dorsal surface of propodeum and declivity separated by roundly-angled posterodorsal corner (Fig. 10B)	**14**
11	Smaller species; TL < 4 mm (Fig. 11A); HW < 0.80 mm	**12**
–	Larger species; TL > 5 mm (Fig. 11B); HW > 0.80 mm	**13**
12	Clypeal median lobe relatively long; frontal carina relatively long, reaching anterior to mid-length of head; body entirely yellowish brown (Thailand)	***B.troglomorpha* Duanchay & Jaitrong**
–	Clypeal median lobe relatively short; frontal carina relatively short, reaching level posterior margin of eye; body entirely black	***B.brevidorsa* Xu**
13	In full-face view, outline between mandibular base and anterior margin of eye (malar space) strongly convex (Fig. 12A); in lateral view, propodeal declivity rugose at margin; in posterior view propodeal declivity laterally weakly margined, transversely rugose except for median smooth area; rugae extending to posterior portion of lateral face of propodeum (Fig. 12B); body color brownish yellow (New Guinea, Java)	***B.arcuata* (Karavaiev)**
–	In full-face view, outline between mandibular base and anterior margin of eye (malar space) nearly straight (Fig. 12C); in lateral view, propodeal declivity smooth at margin; in posterior view propodeal declivity smooth (Fig. 12D); body color black to brownish black (China)	***B.paraarcuata* Chen, Yu & Yi, sp. nov.**
14	Large species, TL > 4.5 mm, HW > 0.9 mm; antennal segments 3 and 4 each longer than broad (Fig. 13A, B); body color usually dark brown	**15**
–	Small-sized species, TL < 4.5 mm, HW < 0.9 mm; antennal segments 3 and 4 each as long as broad, or broader than long (Fig. 13C, D); body color usually yellowish brown to tan	**19**
15	Petiolar node thick (PL > 0.40 mm); in dorsal view, petiole long trapezoidal shape (Fig. 14A) (China)	***B.candida* Chen, Yu & Yi, sp. nov.**
–	Petiolar node thin (PL < 0.40 mm); in dorsal view, petiole semicircular or short trapezoid (Fig. 14B-D)	**16**
16	Antennal scape shorter, surpassing posterior margin of head by < 1/4 of its length; legs yellowish brown to orangish, contrasting with jet black mesosoma (Fig. 15A) (Myanmar)	***B.flavipes* (Yamane)**
–	Antennal scape longer, surpassing posterior margin of head by > 1/4 of its total length; legs brown to dark brown, contrast with body color weaker (Fig. 15B)	**17**
17	Standing hairs on mesosomal dorsum absent or very few; standing hairs if any generally shorter than width of antennal segment 3 (Fig. 16A) (Sumatra)	***B.batak* (Yamane)**
–	Mesosomal dorsum usually with > 10 standing hairs, some of which are as long as or longer than width of antennal segment 3 (Fig. 16B)	**18**
18	Mesopleuron often with transverse groove; groove sometimes incomplete but at least scar visible (Fig. 17A); posterior faces of propodeum and petiole more strongly punctate (Fig. 17C); gastral tergite 1 usually with > 10 standing hairs (Fig. 17C) (Borneo; Malay Peninsula, Java)	***B.pilidorsalis* (Yamane)**
–	Mesopleuron usually without such groove; groove if any vestigial (Fig. 17B); posterior face of propodeum medially and petiole entirely smooth or very weakly punctate (Fig. 17D); gastral tergite 1 with fewer standing hairs, the number, excluding those on posterior margin of tergite, being usually less than ten (Fig. 17D) (China, Philippines, Vietnam, Thailand, Myanmar, Sumatra, India, Nepal)	***B.nigrita* (Emery)**
19	In dorsal view, gastral tergite 1 with densely sub-decumbent hairs (Fig. 18A, B)	**20**
–	In dorsal view, gastral tergite 1 with sparsely sub-decumbent hairs or fewer standing hairs (Fig. 18C, D)	**21**
20	Eye small, with maximum width of scape accounting for 83% of the longest axis of eye; body color reddish brown (Fig. 19A) (Vietnam, Sri Lanka, India)	***B.jerdonii* (Forel)**
–	Eye moderately large, with maximum width of scape accounting for 65% of the longest axis of eye; body color tan (Fig. 19B) (Christmas Island)	***B.christmasi* (Donisthorpe)**
21	In lateral view, dorsal surface of propodeum convex (Fig. 20A) (China, Vietnam, Laos, Thailand, Myanmar, India, Sri Lanka, Sumatra, Philippines, Borneo, Sulawesi, Java)	***B.luteipes* (Mayr)**
–	In lateral view, dorsal surface of propodeum concave or almost straight (Fig. 20B, C)	**22**
22	Upper part of propodeum with sparsely sub-decumbent hairs; body color reddish brown (Fig. 20B) (Australia, New Guinea)	***B.croceicornis* (Emery)**
–	Upper part of propodeum with densely sub-decumbent hairs; body color dark brown (Fig. 20C) (China, Philippines, Borneo, Malaysia, Singapore, India, Sri Lanka)	***B.obscurans* (Walker)**

**Figure 2.**
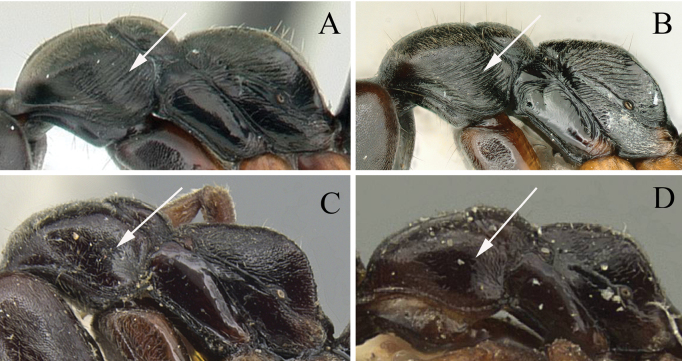
Mesosoma in profile showing sculpture of lateral face of pronotum **A***Brachyponeraatrata* (CASENT0178452) **B***B.wallacea* (non-type) **C***B.chinensis* (CASENT0903937, type) **D***B.luteipes* (CASEN0915672, type). Photographers April Nobile (**A**), Chao Chen (**B**), Will Ericson (**C**), Harald Bruckner (**D**).

**Figure 3.**
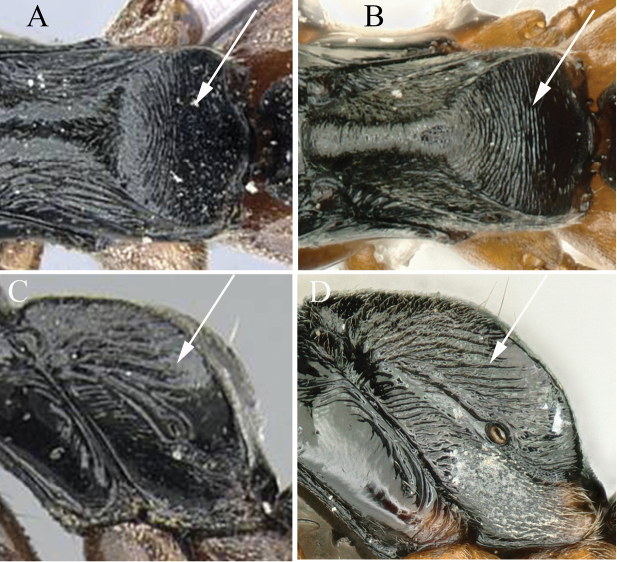
Mesosoma in dorsal view **A***Brachyponeraatrata* (CASENT0178452) **B***B.wallacea* (non-type) Propodeum in lateral view **C***Brachyponeraatrata* (CASENT0178452) **D***B.wallacea* (non-type). Photographers April Nobile (**A, C**), Chao Chen (**B, D**).

**Figure 4.**
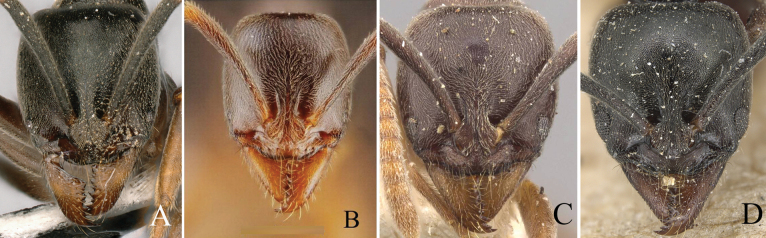
Head in full-face view showing size of eye **A***Brachyponeramyops* sp. nov. **B***B.kumtongi***C***B.jerdonii* (CASENT0907282, type) **D***B.nigrita* (CASENT0903936, type). Photographers Chao Chen (**A**), Duanchay et al. 2024 (**B**), Z. Liebermantype (**C**), Will Ericson (**D**).

**Figure 5.**
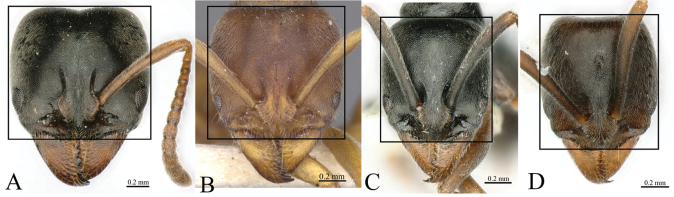
Shape of head in full-face view **A***Brachyponerasennaarensis***B***B.lutea* (CASENT0902499, type) **C***B.nakasujii***D***B.arcuata*. Photographers Chao Chen (**A, C, D**), Will Ericson (**B**).

**Figure 6.**
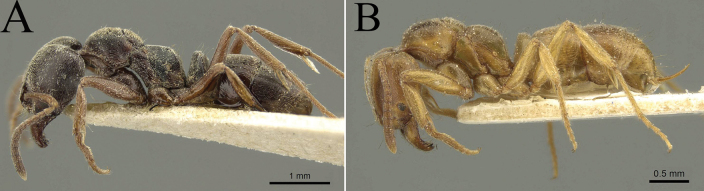
Body color in lateral view **A***Brachyponerasennaarensis* (CASENT0915669, type) **B***B.lutea* (CASENT0915668, type). Photographer Harald Bruckner (**A, B**)

**Figure 7.**
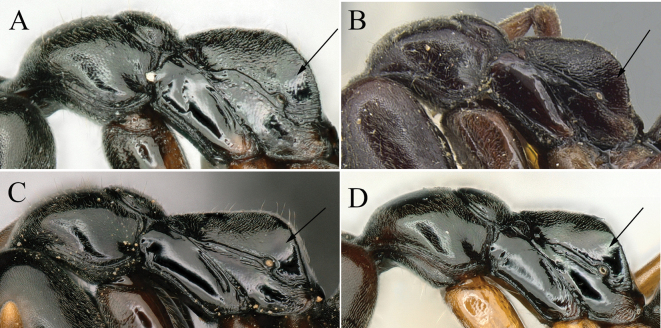
Mesosoma in profile showing sculpture of propodeum **A***Brachyponeranakasujii***B***B.chinensis* (CASENT0903937, type) **C***B.candida* sp. nov. **D***B.flavipes*. Photographers Chao Chen (**A, C, D**), Will Ericson (**B**).

**Figure 8.**
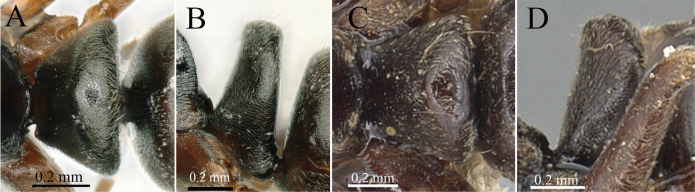
Petiole node in dorsal view and in lateral view **A**, **B***Brachyponeranakasujii* (non-type) **C**, **D***B.chinensis* (CASENT0903937, type). Photographers Chao Chen (**A, B**), Will Ericson (**C, D**).

**Figure 9.**
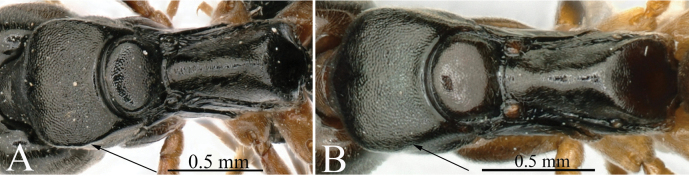
Mesosoma in dorsal view **A***Brachyponeraxui* sp. nov. **B***B.batak.* Photographer Chao Chen (**A, B**).

**Figure 10.**
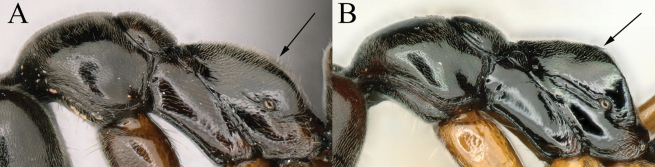
Shape of propodeum in lateral view **A***Brachyponeraparaarcuata* sp. nov. **B***B.flavipes*. Photographer Chao Chen (**A, B**).

**Figure 11.**
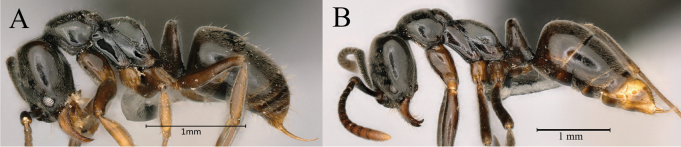
Habitus in lateral view, showing body length **A***Brachyponerabrevidorsa***B***B.paraarcuata* sp. nov. Photographer Chao Chen (**A, B**).

**Figure 12.**
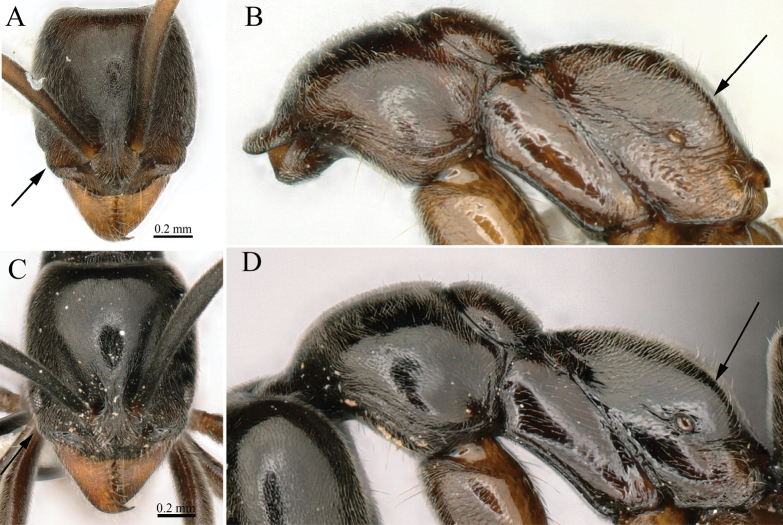
**A**, **C** head in full-face view **B**, **D** mesosoma in lateral view **A**, **B***Brachyponeraarcuate* (non-type from Java) **C**, **D***B.paraarcuata* sp. nov. Photographer Chao Chen (**A–D**).

**Figure 13.**
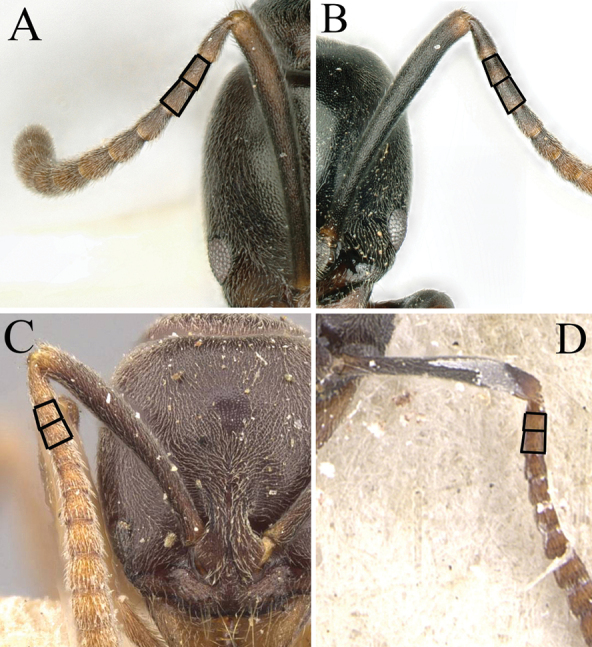
Shape of antennal segment in full-face view **A***Brachyponerabatak* (non-type) **B***B.candida* sp. nov. **C***B.jerdonii* (CASENT0907282, type) **D***B.obscurans* (CASENT0902495, type). Photographers Chao Chen (**A, B**), Z. Liebermantype (**C**), Will Ericson (**D**).

**Figure 14.**
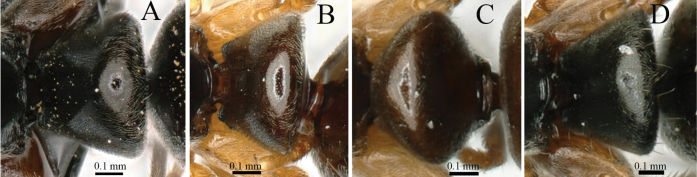
Petiolar node in dorsal view **A***Brachyponeracandida* sp. nov. **B***B.flavipes* (paratype) **C***B.batak*. (non-type) **D***B.pilidorsalis* (non-type). Photographer Chao Chen (**A–D**).

**Figure 15.**
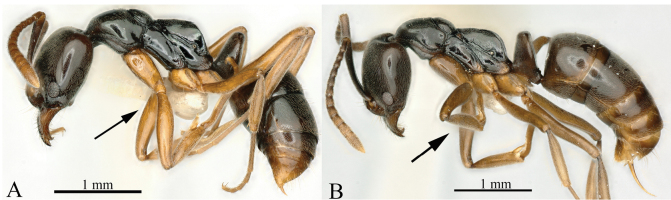
Legs color in lateral view **A***Brachyponeraflavipes* (paratype) **B***B.batak* (non-type). Photographer Chao Chen (**A, B**).

**Figure 16.**
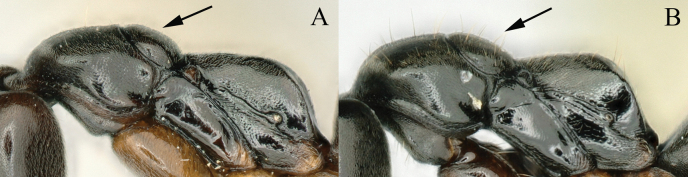
Mesosoma in lateral view **A***Brachyponerabatak* (non-type) **B***B.pilidorsalis* (non-type from West Malaysia). Photographer Chao Chen (**A, B**).

**Figure 17.**
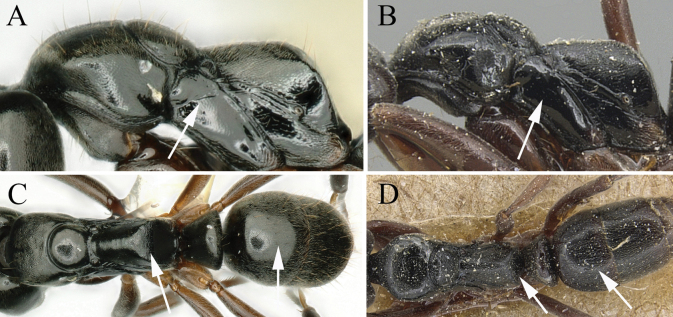
**A**, **B** mesosoma in lateral view **C**, **D** body in dorsal view **A**, **C***Brachyponerapilidorsalis* (non-type from West Malaysia) **B**, **D***B.nigrita* (CASENT0903936, type). Photographers Chao Chen (**A, C**), Will Ericson (**B, D**).

**Figure 18.**
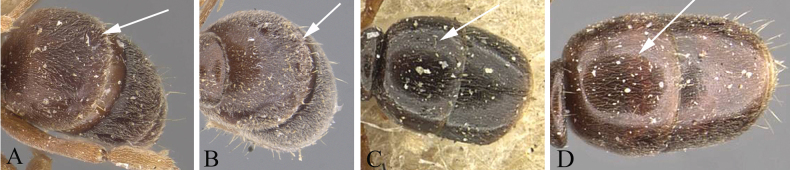
Gastral in dorsal view **A***Brachyponerajerdonii* (CASENT0907282, type) **B***B.christmasi* (CASENT0902496, type) **C***B.luteipes* (CASENT0915672, type) **D***B.croceicornis* (CASENT0907286, type). Photographers Z. Liebermantype (**A, D**), Will Ericson (**B**), Harald Bruckner (**C**).

**Figure 19.**
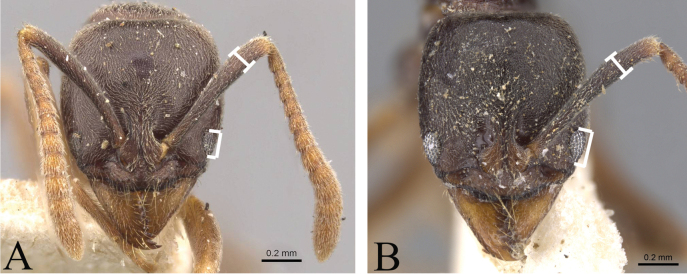
Head in full-face view **A***Brachyponerajerdonii* (CASENT0907282, type) **B***B.christmasi* (CASENT0902496, type). Photographers Z. Lieberman (**A**), Will Ericson (**B**).

**Figure 20.**
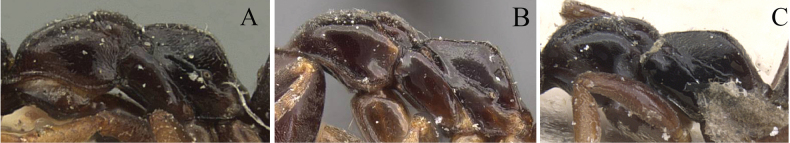
Mesosoma in lateral view **A***Brachyponeraluteipes* (CASENT0915672 type) **B***B.croceicornis* (CASENT0907286, type) **C***B.obscurans* (CASENT0902495, type). Photographers Harald Bruckner (**A**), Z. Lieberman (**B**), Will Ericson (**C**).

### ﻿Revision of the Chinese species of *Brachyponera*

#### ﻿ Brachyponera brevidorsa

Taxon classificationAnimaliaHymenopteraFormicidae

Xu, 1994

4A25D8D0-78F2-542E-966B-ACFF116DB983

Fig. 21A-C

Brachyponera brevidorsa Xu, 1994b: 183, figs 5, 6 (w.). Type locality: China (Yunnan).

##### Type material.

***Holotype*** (worker). China: • Yunnan, Baoshan City, 1800 m, 11.x. 1991, Zhenghui Xu leg (SWFU), No. A91-1022. ***Paratypes*** (workers). China: • 11 workers; same collection data as for holotype.

##### Other material examined.

5 workers; China, • Yunnan, Lvchun City, 1515 m, 24 Jun. 2023, Chao Chen leg., No. KIZ20231057.1-KIZ20231057.5.

##### Measurements and indices.

**Figure 21.**
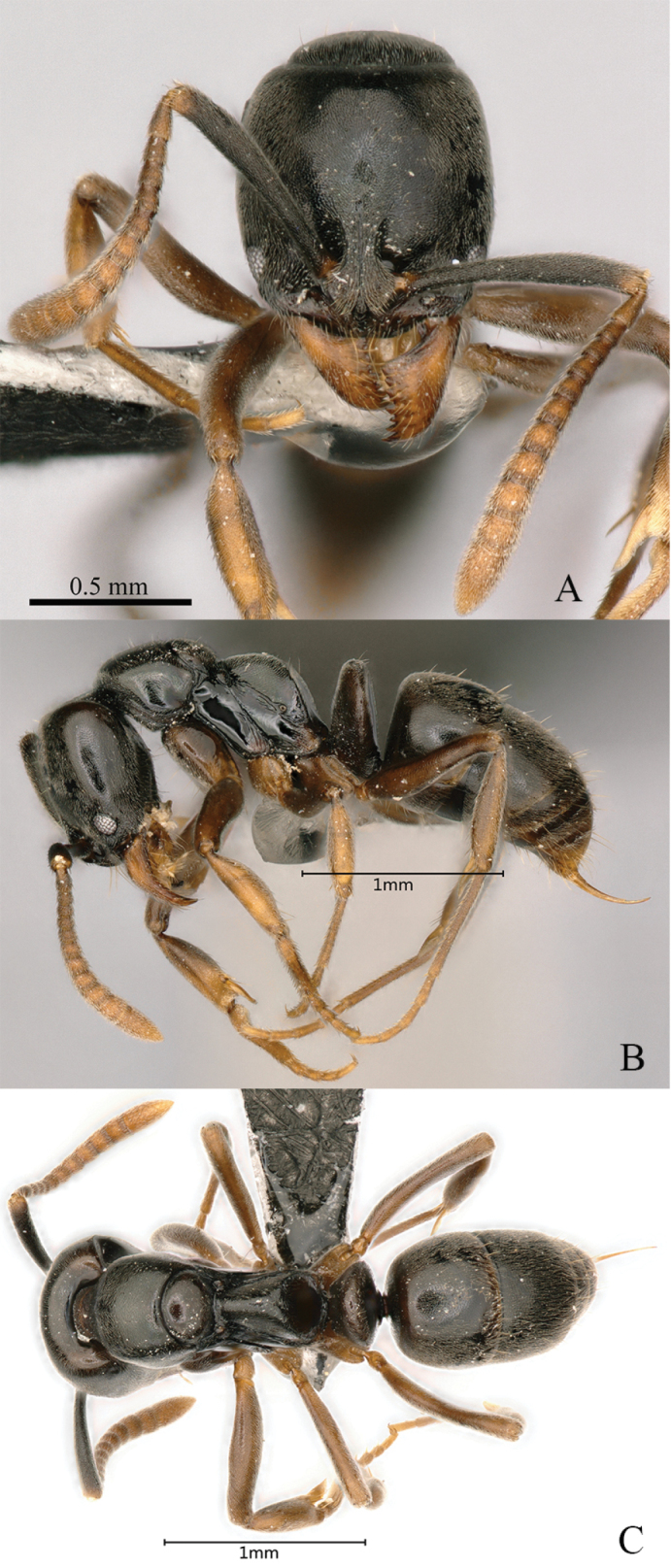
*Brachyponerabrevidorsa* worker (non-type, No. KIZ20231057) **A** head in full-face view **B** body in lateral view **C** body in dorsal view. Photographer Chao Chen.

Worker (Fig. 21A–C): HL 0.84, HLL 0.77, HLA 0.11, HW 0.80, ML 0.46, CML 0.09, CI 95, SL 0.73, SI 92, ED 0.11, PrL 0.48, PrH 0.42, PrW 0.58, WL 1.16, TL 3.8, PL 0.26, PH 0.59, DPW 0.42, LPI 229, PDPI 165.

##### Diagnosis.

The species can be separated from other Chinese members of the genus based on the combination of the following characters: smaller species, < 4 mm in total body length; antenna scape short, slightly exceeds posterolateral corner; clypeal median lobe relatively short; frontal carina relatively short, reaching level posterior margin of eye; in lateral view, pronotum relatively flat; propodeum low, dorsum slightly raised, obviously shorter than declivity, transition between declivity and dorsum is rounded; body entirely black.

##### Brief description.

In full-face view, head longer than broad, roughly trapezoidal, posterolateral corners narrowly rounded. Mandible triangular with nine teeth. Antennae 12-segmented, scape slightly exceeds posterolateral corner, flagellar segments gradually become thicker and rod-shaped towards the end. Eye medium in size and located in front of lateral margin of head. In lateral view, promesonotal suture depressed, metanotal groove deeply impressed. Propodeum convex, mesonotum prominently elevated. Propodeum low, dorsum slightly raised, obviously shorter than declivity, transition between declivity and dorsum is rounded. Petiolar node nearly trapezoidal; subpetiolar process wedge-shaped. In dorsal view, propodeum anterior part narrower than posterior part. Petiolar node crescent-shaped. Head densely punctate. Pronotum with densely punctured. Propodeum lateral margin shiny; dorsum of mesonotum and propodeum shiny. dorsal surface of body with sparsely erect or suberect hairs and densely sub-decumbent hairs. Body color black, mandible, flagellum, legs, and end of gaster yellowish brown.

#### ﻿ Brachyponera candida

Taxon classificationAnimaliaHymenopteraFormicidae

Chen, Yu & Yi sp. nov.

1022AC35-7510-502A-97C3-DB98EC71081C

https://zoobank.org/6CE2B706-123A-4270-89BB-4D3C03C122BA

Fig. 22A-C

##### Type material.

***Holotype***: worker, China: • Yunnan Province, Honghe Hani and Yi Autonomous Prefecture, Lvchun County, Banpo Township, mohelongtang, 22.61721'N, 102.33585'E, 1263 m above sea level, from mixed coniferous-broad forest, 13.iv.2023, Chao Chen leg., No. KIZ20230168 (KIZ). ***Paratypes***: • 5 workers, same data as holotype (KIZ20230168A, KIZ20230168B deposited at SWFU; KIZ20230168C, KIZ20230168D, KIZ20230168E deposited at GXNU).

##### Non-type material examined.

16 workers, China: • Yunnan Province, Honghe Hani and Yi Autonomous Prefecture, Lvchun County, Banpo Township, mohelongtang, 22.61721'N, 102.33585'E, 1263 m above sea level, from mixed coniferous-broad forest, 13.iv.2023, Chao Chen leg., No. KIZ20230168.1-KIZ20230168.16 (KIZ); • 1 worker, Honghe Hani and Yi Autonomous Prefecture, Lvchun County, Banpo Township, Erfu Village, 22.62882'N, 102.35430'E, 1497 m Monsoon evergreen broad-leaved forest, 13.iv.2023, Chao Chen leg., No. KIZ20230194 (KIZ); 1 worker, Honghe Hani and Yi Autonomous Prefecture, Lvchun County, Qimaba Township, Xiaoheijiang, 22.69476'N, 102.33646'E, 510 m Tropical seasonal rainforest, 13.iv.2023, Qiwei Shen leg., No. KIZ20230373 (KIZ); • 1 worker, Honghe Hani and Yi Autonomous Prefecture, Lvchun County, Banpo Township, Bayanhongdong, 22.63598'N, 102.34737'E, 1258 m Monsoon evergreen broad-leaved forest, 17.iv.2023, Huiping Zeng leg., No. KIZ20230502 (KIZ).

**Figure 22.**
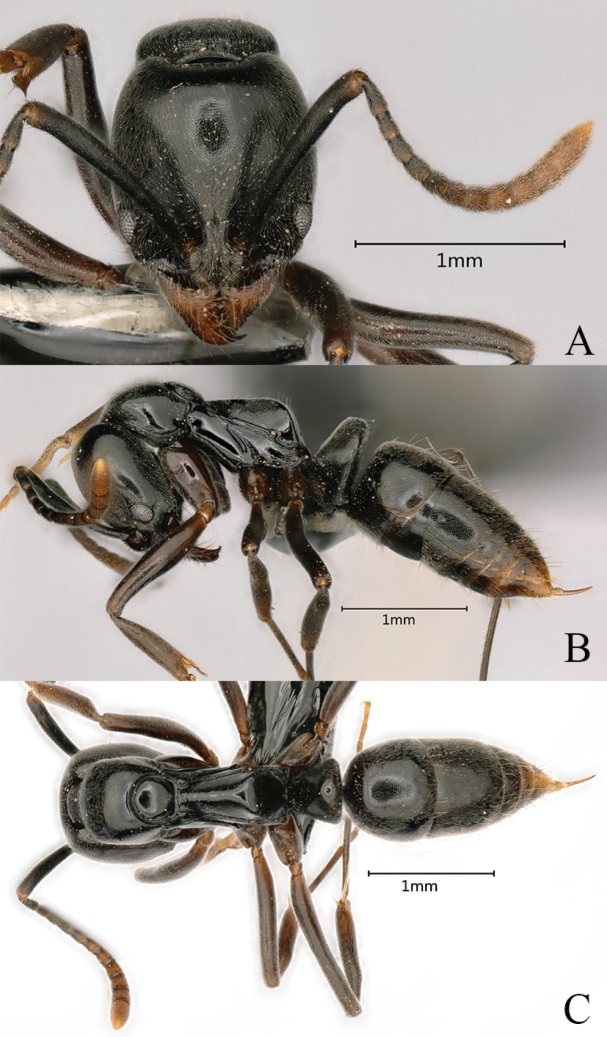
*Brachyponeracandida* sp. nov. worker (holotype, No. KIZ20230168) **A** head in full-face view **B** body in lateral view **C** body in dorsal view. Photographer Chao Chen.

##### Measurements and indices.

***Holotype***: HL 1.07, HLL 0.97, HLA 0.12, HW 0.99, ML 0.52, CML 0.11, CI 93, SL 1.00, SI 101, ED 0.17, PrL 0.64, PrH 0.53, PrW 0.71, WL 1.63, TL 5.2, PL 0.43, PH 0.75, DPW 0.53, LPI 174, PDPI 123. ***Paratypes*** (*n* = 5): HL 1.04–1.11, HLL 0.92–0.97, HLA 0.11–0.19, HW 0.94–1.00, ML 0.50–0.58, CML 0.10–0.12, CI 88–95, SL 0.99–1.09, SI 101–114, ED 0.15–0.17, PrL 0.62–0.67, PrH 0.51–0.57, PrW 0.73–0.79, WL 1.62–1.70, TL 5.0–5.3, PL 0.44–0.47, PH 0.75–0.79, DPW 0.51–0.58, LPI 160–173, PDPI 113–126.

##### Diagnosis.

The new species is similar to *B.pilidorsalis* (Yamane, 2007). However, it can be separated by relatively short scape that exceeds the posterolateral corner by 1/10 of its length (in *B.pilidorsalis* scape is longer, exceeding posterolateral corner with more than ¼ of its length); slightly shorter distance between anterior clypeal margin to anterior margin of torulus, CML 0.11 (relatively long in *B.pilidorsalis*, CML 0.15), dorsal length of propodeum longer than declivity, mesopleuron usually without a groove, dorsum of mesosoma with slightly short erect hairs (dorsal length of propodeum slightly longer than declivity, mesopleuron often with a transverse groove, dorsum of mesosoma with slightly long standing hairs in *B.pilidorsalis*).

##### Description.

In full-face view, head longer than broad, roughly rectangular, posterior margin weakly concave, posterolateral corners narrowly rounded, lateral margins moderately convex. Mandible triangular with eight teeth, apical tooth largest, and with a basal mandibular pit. Clypeus transverse, center of anterior margin moderately concave. Frontal carina short, frontal lobes well developed, covering antennal socket, frontal region with central longitudinal ridge. Antennae 12-segmented, scape 1/10 exceeds posterolateral corner, flagellum gradually increases in size toward the end. Eye medium and maximum diameter consists of eleven ommatidia (ED 0.17 mm).

In lateral view, pronotum and mesonotum significantly higher than propodeum. Promesonotal suture seams evident, metanotal groove deeply impressed. Mesonotum moderately convex. Dorsal surface of propodeum nearly straight, with a length ~ 1.3× that of declivity, slope of declivity steep, nearly straight. Posterodorsal corner of propodeum broadly rounded. Propodeal spiracle rounded, with a groove between spiracle and metanotal groove. Metapleural bulla not visible. Petiolar node as high as propodeum, upright, thick (PDPI 123), nearly trapezoidal; anterior margin and dorsum moderately convex, posterior margin nearly straight; subpetiolar process forms a wedge. Prora absent. Gaster subconical, basal two intersegments contracted, apex with sting.

In dorsal view, pronotum widest in mesosoma, humeral corners bluntly rounded; lateral margins moderately convex. Anterior margin of mesonotum convex, posterior margin slightly straight. Propodeum nearly rectangular, gradually narrowing from bottom to the top, forming a ridge. Petiolar node trapezoidal, front narrow and gradually widening backwards; anterior margin flat, posterior margin moderately convex.

Dorsal surface of body with densely hairy punctation. Mesopleuron, metapleural and lower part of lateral side of petiolar node smooth and shiny. Dorsal surface of body with sparsely erect or suberect hairs and densely sub-decumbent hairs. Body color black, funiculus, mandible, and legs brownish black.

##### Ecological notes.

The new species was collected in the Huanglianshan National Nature Reserve in Yunnan. The type series was collected from a nest in soil, which was built in a coniferous and broad-leaved mixed forest at an altitude of 1250 m. Sixteen workers of this new species were collected foraging on the forest floor. An additional 61 workers of this new species were collected in five sample plots. The forest types include tropical seasonal rainforest, monsoon evergreen broad-leaved forest, and mountain moss evergreen broad-leaved forest. The new species builds its nest in the soil. Workers were found foraging on the ground and tree trunks. The altitude of all sample plots is below 2000 m (Suppl. material 1: fig. S15).

##### Etymology.

The new species name refers to its smooth and shiny body, with only a relatively small number of punctures.

#### ﻿ Brachyponera chinensis

Taxon classificationAnimaliaHymenopteraFormicidae

(Emery, 1895)

E64D60C7-0E96-5DD5-8821-57A1A20D7FB0

Fig. 23A-D

Ponera nigrita subsp. chinensis Emery, 1895 m: 460 (in text) (w.). Type locality: China (Shanghai). Indomalaya.

##### Type material.

***Holotype*** worker, from China, • Shanghai, date and collector unknown (images of the holotype, CASENT0903937 available on AntWeb were examined).

##### Measurements and indices.

**Figure 23.**
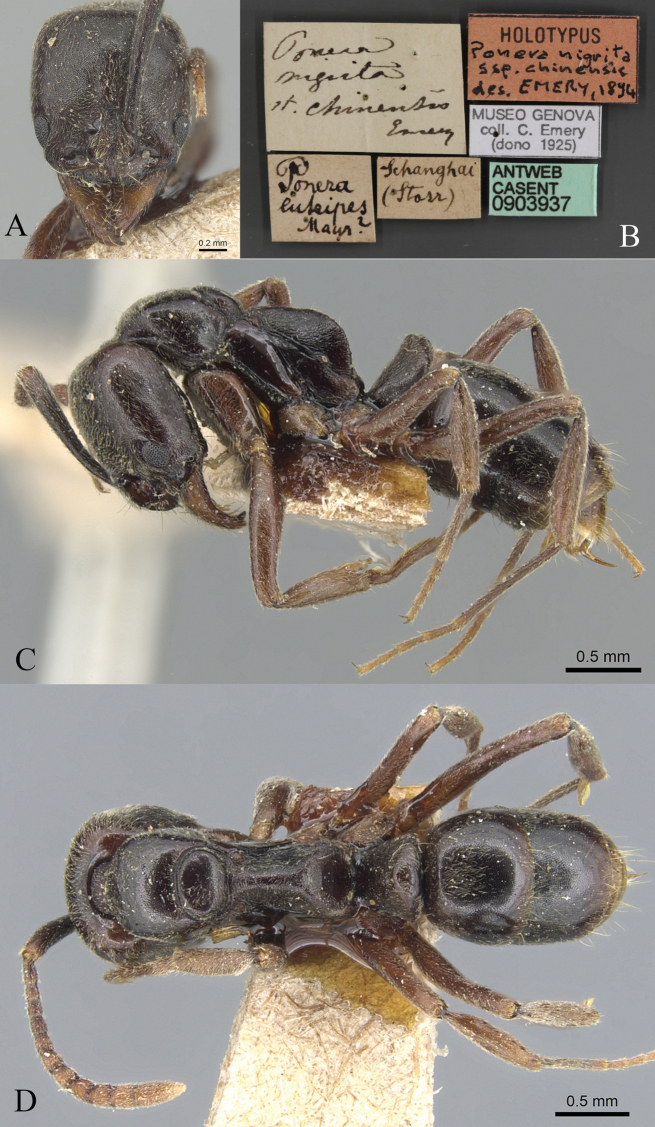
*Brachyponerachinensis* worker (CASENT0903937, type) **A** head in full-face view **B** label **C** body in lateral view **D** body in dorsal view. Photographer Will Ericson.

Worker (Fig. 23A–D): HL 1.26, HLL 1.15, HLA 0.14, HW 1.16, ML 0.69, CML 0.19, CI 92, SL 0.99, SI 86, ED 0.23, PrL 0.71, PrH 0.57, PrW 0.77, WL 1.67, TL 5.0, PL 0.40, PH 0.60, DPW 0.58, LPI 150, PDPI 145.

##### Diagnosis.

The species can be separated from other Chinese members of the genus based on the combination of the following characters: mandible base rough with fine puncta, tip smooth; antennae 12-segmented, scape slightly exceeds posterolateral corner; eye medium in size and located in front of lateral margin of head; in lateral view, propodeum densely transversely rugose; smooth area if any much restricted; dorsal of propodeum straight, declivity convex, the length is nearly equal.

##### Brief description.

In full-face view, head longer than broad, roughly trapezoidal, posterolateral corners narrowly rounded. Mandible triangular with nine teeth. Antennae 12-segmented, scape slightly exceeds posterolateral corner, flagellar segments gradually become thicker and rod-shaped towards the end. Eye medium in size and located in front of lateral margin of head. In lateral view, metanotal groove deeply impressed. Dorsum of propodeum straight, declivity convex, the length is nearly equal. Petiolar node nearly trapezoidal; subpetiolar process wedge-shaped. In dorsal view, pronotum anterior and lateral margins broad and round, posterior margin concave. Petiolar node crescent-shaped. Mandible base rough with fine puncta, tip smooth. Head and body densely and finely punctate; pronotum, petiolar node and gaster with fine, shiny puncta. Side plates of mesosoma smooth and shiny. Propodeum rough with dense, coarse puncta. Body black; mandible, antennae flagellum, legs, and end of gaster yellow brown.

#### ﻿ Brachyponera luteipes

Taxon classificationAnimaliaHymenopteraFormicidae

(Mayr, 1862)

DD5B5388-95DE-566E-B927-5F24111D65F5

Fig. 24A-D

Ponera luteipes Mayr, 1862: 722 (w.q.), India (Nicobar Is).

##### Type material.

Syntype workers and syntype queens (NHMW) from India, Nicobar Is, Milu I. (images of a syntype, CASENT0915672, available on AntWeb were examined).

##### Non-type material examined.

• 13 workers, China: Yunnan Province, Honghe Hani and Yi Autonomous Prefecture, Lvchun County, Banpo Township, Daguyan, 22.61094'N, 102.28100'E, 367 m Tropical seasonal rainforest, 9.x.2023, Chao Chen leg., No. KIZ20231066.1- KIZ20231066.13(KIZ); • 20 workers, Honghe Hani and Yi Autonomous Prefecture, Lvchun County, Sanmeng Township, Laobianduan, 22.92752'N, 102.28553'E, 1749 m montane mossy evergreen broad-leaf forest, 16.x.2023, Chao Chen leg., No. KIZ20232340.1- KIZ20232340.20(KIZ); • 7 workers, Honghe Hani and Yi Autonomous Prefecture, Lvchun County, Qimaba Township, 22.80584'N, 102.22848'E, 745 m mixed coniferous and broad-leaved forest, 13.x.2023, Chao Chen leg., No. KIZ20232464.1- KIZ20232464.7(KIZ).

##### Measurements and indices.

**Figure 24.**
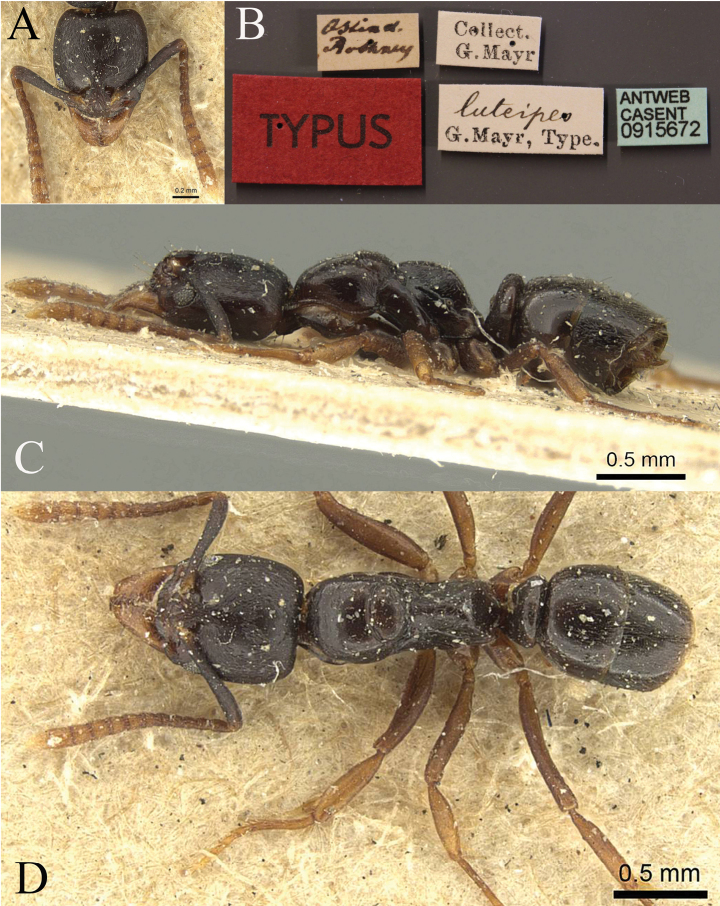
*Brachyponeraluteipes* worker (CASENT0915672, type) **A** head in full-face view **B** label **C** body in lateral view **D** body in dorsal view. Photographer Harald Bruckner.

worker (Fig. 24A–D): HL 0.78, HLL 0.70, HLA 0.10, HW 0.71, ML 0.40, CML 0.08, CI 91, SL 0.65, SI 92, ED 0.11, PrL 0.45, PrH 0.32, PrW 0.50, WL 1.04, TL 3.0, PL 0.21, PH 0.44, DPW 0.39, LPI 211, PDPI 186.

##### Diagnosis.

The species can be separated from other Chinese members of the genus based on the combination of the following characters: smaller species, < 4.5 mm in total body length; head width < 0.9 mm; flagellar segments 1 and 2 each as long as broad, or broader than long; dorsum of propodeum convex, declivity convex, the length is nearly equal; in lateral view, propodeum smooth or only punctate; in dorsal view, gastral tergite 1 with sparsely sub-decumbent hairs or fewer standing hairs.

##### Brief description.

Head longer than wide, with broad rounded depression at posterior margin. Mandible triangular with nine teeth. Antennae 12-segmented, scape exceeds posterolateral corner, flagellar segments 1 and 2 as long as broad, or broader than long. Dorsum of propodeum convex, declivity convex, the length is nearly equal Mandible smooth, finely punctate. Head and body densely and finely punctate; pronotum and mesonotum puncta are rough; side plates of mesosoma smooth and shiny; Petiolar node and gaster finely punctate. Dorsal surface of head and body sparsely erected with abundant sub-decumbent hairs; scape and hind tibiae are densely covered in sub-decumbent hairs, lacking erected hairs. Body black; mandible, antennae flagellum, tibiae, tarsus, and end of gaster reddish brown.

#### ﻿ Brachyponera myops

Taxon classificationAnimaliaHymenopteraFormicidae

Chen, Yu & Yi sp. nov.

401EC2C6-4214-5903-A665-9E2277EE722B

https://zoobank.org/C195D65F-5790-4B18-9705-577A18240D26

Fig. 25A–C

##### Type material.

***Holotype***: worker, China: • Yunnan Province, Honghe Hani and Yi Autonomous Prefecture, Lvchun County, Niukong Town, niaoliuyaozhai, 22.95837'N, 102.28872'E, 1515 m above sea level, *Eucalyptus* forest (plantation), 24.iv.2023, Chao Chen leg., No. KIZ20231049 (KIZ). ***Paratypes***: • 1 worker (KIZ20231049A), same data as holotype (SWFU); 4 workers (KIZ20230651, KIZ20230651A, KIZ20230651B, KIZ20230651C): Yunnan Province, Honghe Hani and Yi Autonomous Prefecture, Lvchun County, Qimaba Township, Omo village, 22.68445'N, 102.29683'E, 1518 m above sea level, from monsoon evergreen broad-leaved forest, 18.iv.2023, Huiping Zeng leg.(GXNU).

##### Non-type material examined.

• 4 workers, China: Yunnan Province, Honghe Hani and Yi Autonomous Prefecture, Lvchun County, Qimaba Township, Omo village, 22.68445'N, 102.29683'E, 1518 m above sea level, from monsoon evergreen broad-leaved forest, 18.iv.2023, Huiping Zeng leg., No. KIZ20230647, KIZ20230654.1- KIZ20230654.3 (KIZ); • 18 workers, Honghe Hani and Yi Autonomous Prefecture, Lvchun County, Niukong Township, niaoliuyaozhai, 22.95837'N, 102.28872'E, 1515 m above sea level, *Eucalyptus* forest (plantation), 24.iv.2023, Chao Chen leg., No. KIZ20231046.1-20231046.18 (KIZ).

##### Measurements and indices.

***Holotype***: HL 1.09, HLL 0.98, HLA 0.18, HW 1.00, ML 0.61, CML 0.13, CI 92, SL 1.04, SI 104, ED 0.07, PrL 0.65, PrH 0.57, PrW 0.72, WL 1.58, TL 5.0, PL 0.40, PH 0.75, DPW 0.50, LPI 188, PDPI 125. ***Paratype*** (*n* = 5): HL 0.99–1.08, HLL 0.90–0.95, HLA 0.12–0.15, HW 0.90–0.98, ML 0.62–0.69, CML 0.10–0.14, CI 90–96, SL 0.98–1.1, SI 99–112, ED 0.06–0.07, PrL 0.62–0.68, PrH 0.50–0.57, PrW 0.74–0.79, WL 1.51–1.59, TL 4.9–5.3, PL 0.41–0.48, PH 0.71–0.76, DPW 0.45–0.48, LPI 148–185, PDPI 96–117.

##### Diagnosis.

The new species is similar to *Brachyponeraluteipes* (Mayr, 1862), but it differs in scape segments 3–7 longer than wide, small eye with maximum diameter consisting of five ommatidia (ED 0.07 mm), and inconspicuous or absent groove between spiracle and metanotal groove. In *B.luteipes*, scape segments 3–7 are subsquare, eye is moderately big with maximum diameter consisting of nine ommatidia (ED 0.13 mm), and groove between spiracle and metanotal groove is conspicuous.

**Figure 25.**
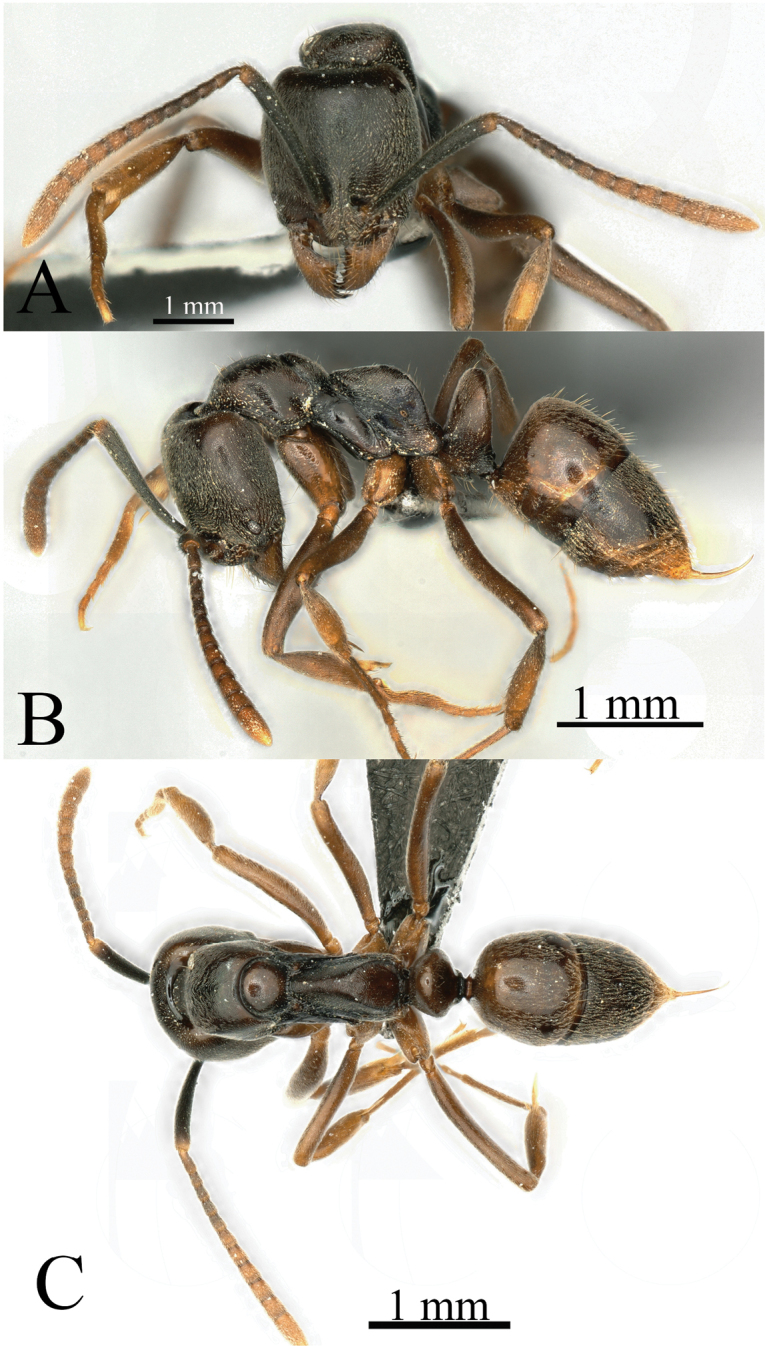
*Brachyponeramyops* sp. nov. worker (holotype No. KIZ20231049) **A** head in full-face view **B** body in lateral view **C** body in dorsal view. Photographer Chao Chen.

##### Description.

In full-face view, head longer than broad, roughly trapezoidal, posterior margin weakly concave, posterolateral corners narrowly rounded, lateral margins moderately convex. Mandible triangular with nine teeth, end tooth largest, and with a basal mandibular pit. Clypeus transverse, center of anterior margin moderately concave. Frontal carina short, frontal lobes well developed, covering antennal socket, frontal region with central longitudinal ridge. Antennae 12-segmented, scape exceeds posterolateral corner of head by 1/5 of its length, flagellar segments gradually increase in size toward the end. Eye small, and maximum diameter consists of five ommatidia (ED 0.07 mm).

In lateral view, anterior margin of pronotum forms a cervical shield extending forward. Pronotum and mesonotum significantly higher than propodeum. Promesonotal suture seams evident, metanotal groove deeply impressed. Mesonotum moderately convex. Dorsal surface of propodeum weakly convex, slightly longer than declivity, slope of declivity steep, nearly convex; posterodorsal corner of propodeum broadly rounded. Propodeal spiracle rounded, groove between spiracle and metanotal groove inconspicuous or absent. Metapleural bulla small, elliptical. Petiolar node as high as propodeum, upright, nearly trapezoidal; anterior margin and dorsum moderately convex, posterior margin nearly straight; subpetiolar process wedge. Prora absent. Gaster subconical, basal two intersegments contracted, apex with sting.

In dorsal view, pronotum widest in mesosoma, humeral corners bluntly rounded; lateral margins moderately convex. Mesonotum elliptical. Propodeum nearly rectangular, gradually narrowing from bottom to the top, forming a ridge. Petiolar node trapezoidal, front narrow and gradually widening backwards; anterior margin flat, posterior margin moderately convex.

Mandible with a row of hairy pits. Dorsal surface of body with densely hairy punctation. Mesopleuron and metapleural with longitudinally rugose. dorsal surface of body with sparsely erect or suberect hairs and densely sub-decumbent hairs. Body color brownish black, funiculus, mandible and legs yellowish brown.

##### Ecological notes.

The new species was collected in the Huanglianshan National Nature Reserve, Yunnan. The holotype and one paratype were collected in the same soil nest in a *Eucalyptus* forest (plantation) at an altitude of 1500 m, and four paratypes were found in a monsoon broad-leaved evergreen forest at an altitude of 1500 m. In addition, 22 workers of this new species were found to be nesting in the soil and foraging on forest floor (Suppl. material 1: fig. S17).

##### Etymology.

The specific name refers to the relatively minute size of the compound eye that characterizes workers of this species.

#### ﻿ Brachyponera nigrita

Taxon classificationAnimaliaHymenopteraFormicidae

(Emery, 1895)

FFAB915C-DCFA-5C3C-B9AF-512AEC33845F

Fig. 26A–D

Ponera nigrita Emery, 1895 m: 459 (w.) Myanmar.

##### Type material.

• Syntype workers, Myanmar (“Burma”), Carin Cheba, 500–1500 m., ii.-iii.1888 (L. Fea) (images of a syntype worker, CASENT0903936 available on AntWeb were examined).

##### Measurements and indices.

**Figure 26.**
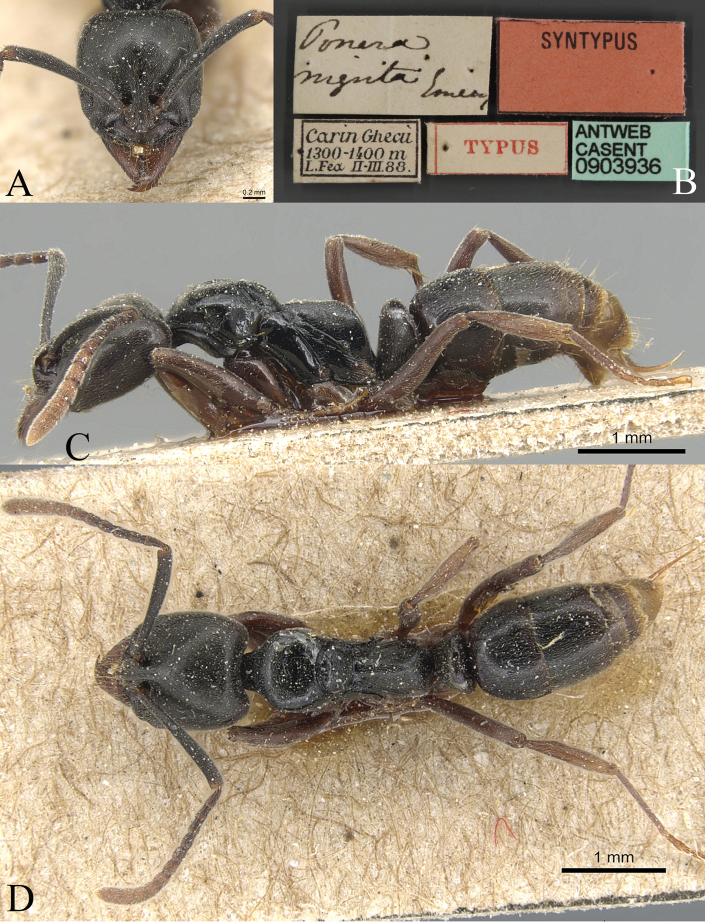
*Brachyponeranigrita* worker (CASENT0903936, type) **A** head in full-face view **B** label **C** body in lateral view **D** body in dorsal view. Photographer Will Ericson.

Worker (Fig. 26A–D): HL 1.23, HLL 1.13, HLA 0.15, HW 1.17, ML 0.72, CML 0.19, CI 95, SL 1.24, SI 106, ED 0.24, PrL 0.83, PrH 0.71, PrW 0.83, WL 2.00, TL 6.0, PL 0.48, PH 0.88, DPW 0.63, LPI 182, PDPI 131.

##### Diagnosis.

The species can be separated from other Chinese members of the genus based on the combination of the following characters: larger species, > 4.5 mm in total body length; head width > 0.9 mm; flagellar segments 1 and 2 longer than broad; posterior face of propodeum medially and petiole entirely smooth or very weakly punctate; gastral tergite 1 with fewer standing hairs, the number, excluding those on posterior margin of tergite, being usually < 10; body color usually dark brown.

##### Brief description.

In full-face view, head longer than broad, roughly trapezoidal. Mandible triangular with nine teeth. Frontal carina short, frontal lobes well developed, covering antennal socket. Antennae 12-segmented, scape exceeds posterolateral corner, flagellar segments gradually increase in size toward the end. Eye medium in size and located in front of lateral margin of head. In lateral view, metanotal groove deeply impressed. Dorsal of propodeum nearly straight, longer than declivity; posterodorsal corner of propodeum broadly rounded. Petiolar node as high as propodeum, upright, nearly trapezoidal; subpetiolar process forms a wedge. In dorsal view, pronotum anterior and lateral margins broad and round, posterior margin concave. Petiolar node crescent-shaped. Mandible densely punctate; head densely punctate; mesosoma smooth, pronotum finely punctate, and propodeum finely rugose; petiolar node and posteroventral area densely finely punctate. Body dorsally with abundant standing hairs and densely sub-decumbent hairs. Body black, appendages yellowish brown.

#### ﻿ Brachyponera obscurans

Taxon classificationAnimaliaHymenopteraFormicidae

(Walker, 1859)

684F95A1-5477-5ACB-9234-E5C9ED028D03

Fig. 27A–D

Formica obscurans Walker, 1859: 372 (q.) Sri Lanka.

##### Type material.

type worker, Primary type locality: Sri Lanka (Ceylon). (images of a syntype worker, CASENT0902495 available on AntWeb were examined).

##### Measurements and indices.

Worker (Fig. 26A–D): HL 0.86, HLL 0.79, HLA 0.10, HW 0.80, ML 0.50, CML 0.14, CI 93, SL 0.80, SI 99, ED 0.16, PrL 0.62, PrH 0.52, PrW 0.56, WL 1.37, TL 4.1, PL 0.30, PH 0.48, DPW 0.48, LPI 159, PDPI 158.

##### Diagnosis.

The species can be separated from other Chinese members of the genus based on the combination of the following characters: smaller species, < 4.5 mm in total body length; head width < 0.9 mm; flagellar segments 1 and 2 each as long as broad, or broader than long; dorsum of propodeum nearly straight, longer than declivity, posterodorsal corner of propodeum broadly rounded; upper part of propodeum with densely sub-decumbent hairs; in dorsal view, petiolar node flat oval; body color usually yellowish brown to tan.

**Figure 27.**
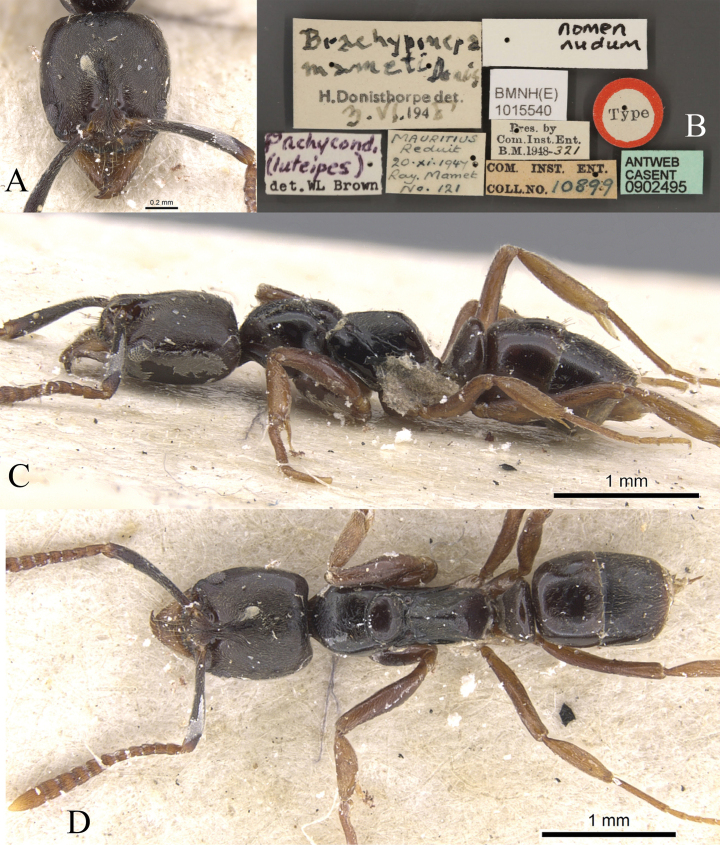
*Brachyponeraobscurans* worker (CASENT0902495, type) **A** head in full-face view **B** label **C** body in lateral view **D** body in dorsal view. Photographer Will Ericson.

##### Brief description.

In full-face view, head longer than broad, roughly trapezoidal, posterolateral corners of head narrowly rounded. Mandible triangular with nine teeth. Antennae 12-segmented, scape exceeds posterolateral corner, flagellar segments gradually increase in size toward the end. Eye medium in size and located in front of lateral margin of head. In lateral view, metanotal groove deeply impressed. Dorsum of propodeum nearly straight, longer than declivity; posterodorsal corner of propodeum broadly rounded. Petiolar node as high as propodeum, upright, nearly trapezoidal; subpetiolar process forms a wedge. In dorsal view, pronotum anterior and lateral margins broad and round, posterior margin concave. Petiolar node flat oval. Mandible densely punctate; head densely punctate; mesosoma, petiole and gaster finely punctate. Dorsum of body with abundant standing hairs and densely sub-decumbent hairs; upper part of propodeum with densely sub-decumbent hairs; body color dark brown, mandible, flagellar segments, and legs deep yellowish brown.

#### ﻿ Brachyponera paraarcuata

Taxon classificationAnimaliaHymenopteraFormicidae

Chen, Yu & Yi sp. nov.

2790AA07-1E53-5B55-BAC2-BF382B7DB3FF

https://zoobank.org/DD7E9165-CB0C-4770-A644-88586152784A

Fig. 28A–C

##### Type material.

***Holotype.*** worker, China: • Yunnan Province, Honghe Hani and Yi Autonomous Prefecture, Lvchun County, Banpo Township, Bayanhongdong, 24.63598'N, 102.34737'E, 1258 m above sea level, from soil in a monsoon evergreen broadleaf forest, 10.X.2023, Bolun Li leg., No. KIZ20231657 (KIZ). ***Paratypes***: • 4 workers, same data as holotype (KIZ20231657A, KIZ20231657B deposited in SWFU, KIZ20231657C, KIZ20231657D deposited in GXNU).

##### Non-type material examined.

1 worker, China: Yunnan Province, Honghe Hani and Yi Autonomous Prefecture, Lvchun County, Banpo Township, mohelongtang, 22.61721'N, 102.33585'E, 1263 m above sea level, from mixed coniferous-broad forest, 13.iv.2023, Chao Chen leg., No. KIZ20230173 (KIZ); 2 workers, Honghe Hani and Yi Autonomous Prefecture, Lvchun County, Banpo Township, Bayanhongdong, 24.63598'N, 102.34737'E, 1258 m above sea level, monsoon evergreen broadleaf forest, 10.X.2023, Bolun Li leg., No. KIZ20230527.1- KIZ20230527.2 (KIZ); 2 workers, Honghe Hani and Yi Autonomous Prefecture, Lvchun County, Qimaba Township, dongma village, 22.68607'N, 102.30688'E, 1267 m monsoon evergreen broad-leaved forest, 18.iv.2023, Chao Chen leg., No. KIZ20230625.1- KIZ20230625.2 (KIZ).

##### Measurements and indices.

**Figure 28.**
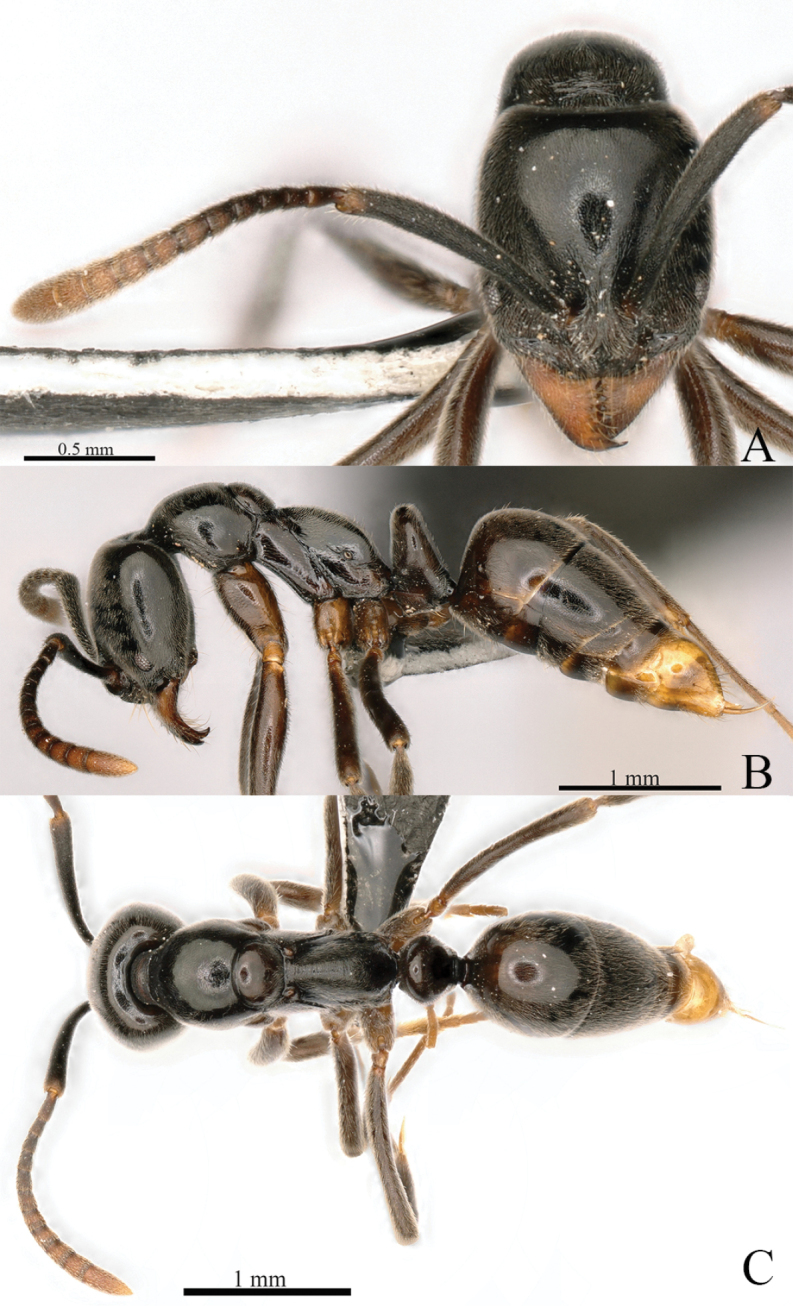
*Brachyponeraparaarcuata* sp. nov. worker (holotype, No. KIZ20231657) **A** head in full-face view **B** body in lateral view **C** body in dorsal view. Photographer Chao Chen.

***Holotype***: (Fig. 28A–C): HL 1.01, HLL 0.93, HLA 0.15, HW 0.91, ML 0.55, CML 0.13, CI 90, SL 0.98, SI 107, ED 0.13, PrL 0.65, PrH 0.54, PrW 0.67, WL 1.51, TL 5.1, PL 0.36, PH 0.68, DPW 0.42, LPI 188, PDPI 117. ***Paratypes*** (n = 4): HL 0.98–1.09, HLL 0.92–0.97, HLA 0.14–0.15, HW 0.90–0.95, ML 0.55–0.57, CML 0.12–0.13, CI 87–92, SL 0.95–0.98, SI 103–105, ED 0.13, PrL 0.60–0.66, PrH 0.54–0.55, PrW 0.61–0.67, WL 1.46–1.51, TL 5.0–5.4, PL 0.34–0.37, PH 0.66–0.68, DPW 0.42–0.43, LPI 184–194, PDPI 116–124.

##### Diagnosis.

The new species is similar to *Brachyponeraarcuata* (Karavaiev, 1925), but it differs in lateral margins of head from mandible base to anterior edge of eye nearly straight (Fig. 12C), propodeal declivity smooth at the margin (Fig. 12D), body color black to brownish black. In *B.arcuata*, lateral margins of head between mandible base and anterior edge of eye are strongly convex (Fig. 12A), propodeal declivity is rugose at margin (Fig. 12B), and body color is brownish yellow.

##### Description.

In full-face view, head longer than broad, roughly rectangular, posterior margin nearly straight, posterolateral corners narrowly rounded, lateral margins moderately convex. Mandible triangular with nine teeth, end tooth largest, and with a basal mandibular pit. Clypeus transverse, center of anterior margin weakly concave. Frontal carina short, frontal lobes well developed, covering antennal socket, frontal region with central longitudinal ridge. Antennae 12-segmented, scape exceeds posterolateral corner of head by 1/5 of its length, flagellar segments gradually increase in size toward the end. Eye medium to small and maximum diameter consists of eight ommatidia (ED 0.13 mm).

In lateral view, anterior margin of pronotum forms a cervical shield extending forward. Pronotum and mesonotum significantly higher than propodeum. Promesonotal suture seams evident, metanotal groove deeply impressed. Dorsal surface of propodeum continuously connected with declivity, forming a complete circular arc. Propodeal spiracle rounded, groove between spiracle and metanotal groove inconspicuous or absent. Metapleural bulla small, roughly elliptical. Petiolar node as high as propodeum, upright, nearly trapezoidal; anterior and posterior margin nearly straight, dorsum moderately convex; subpetiolar process triangular. Prora absent. Gaster subconical, basal two intersegments contracted, apex with sting.

In dorsal view, pronotum broadest, lateral margins moderately convex, humeral corners indistinct. Promesonotal suture and metanotal groove form an ellipse. propodeum rectangular, wide at the lower part and narrow at the upper part. Petiolar node broader than long, lateral and anterior margins convex, posterior margin flat.

Head, pronotum, mesonotum, and gaster with largely densely punctate. Mesopleuron, propodeum, petiolar node and legs with hairy punctations. Clypeus, mandible, coxa, propodeum, petiolar node and gaster with sparse erect or suberect hairs, dorsal surface of body with densely sub-decumbent hairs. Body color black to brownish black, funiculus, mandible, and legs yellowish brown.

##### Ecological notes.

The new species was collected in the Huanglianshan National Nature Reserve in Yunnan. The type series was collected from the same soil nest in a monsoon evergreen broad-leaved forest at an altitude of 1250 m and 14 were collected foraging on the ground surface. One worker was collected in the soil of mixed coniferous and broadleaf forest at an altitude of 1000 m. Two workers were collected on the ground surface of the monsoon evergreen broadleaf forest at an altitude of 1250 m (Suppl. material 1: fig. S18).

##### Etymology.

The specific epithet *paraarcuata* is a compound word meaning “similar to *arcuata*”.

#### ﻿ Brachyponera xui

Taxon classificationAnimaliaHymenopteraFormicidae

Chen, Yu & Yi sp. nov.

31C9FFA1-FB5A-5E7D-80D8-F03EFB3940D3

https://zoobank.org/3AEDBAE9-C634-4790-AAF5-C9AC68B4C014

Fig. 29A–C

##### Type material.

***Holotype*** (worker) China: • Yunnan Province, Honghe Hani and Yi Autonomous Prefecture, Lvchun County, Sanmeng Township, laobianduan, 22.92752'N, 102.28553'E, 1749 m above sea level, from montane mossy evergreen broad-leaved forest, 24.iv.2023, Chao Chen leg., No. KIZ20231023 (KIZ). ***Paratypes***: • 1 worker, same data as holotype, No. KIZ20231004 (SWFU); • 1 worker, Yunnan Province, Honghe Hani and Yi Autonomous Prefecture, Lvchun County, Qimaba Township, Dongma village, 22.69017'N, 102.33054'E, 744 m above sea level, from monsoon evergreen broad-leaved forest, 10.x.2023, Yu Yu leg., No. KIZ20231736 (GXNU).

##### Non-type material examined.

• 2 workers, China: Yunnan Province, Honghe Hani and Yi Autonomous Prefecture, Lvchun County, Qimaba Township, lali village, 22.81959'N, 102.30435'E, 1263 m above sea level, montane mossy evergreen broad-leaf forest, 21.iv.2023, Chao Chen leg., No. KIZ20230837.1-KIZ20230837.2 (KIZ); • 1 worker,, Honghe Hani and Yi Autonomous Prefecture, Lvchun County, Qimaba Township, Xiaoheijiang, 22.69476'N, 102.33646'E, 510 m Tropical seasonal rainforest, 10.x.2023, Bolun Li leg., No. KIZ20231480 (KIZ);

##### Measurements and indices.

**Figure 29.**
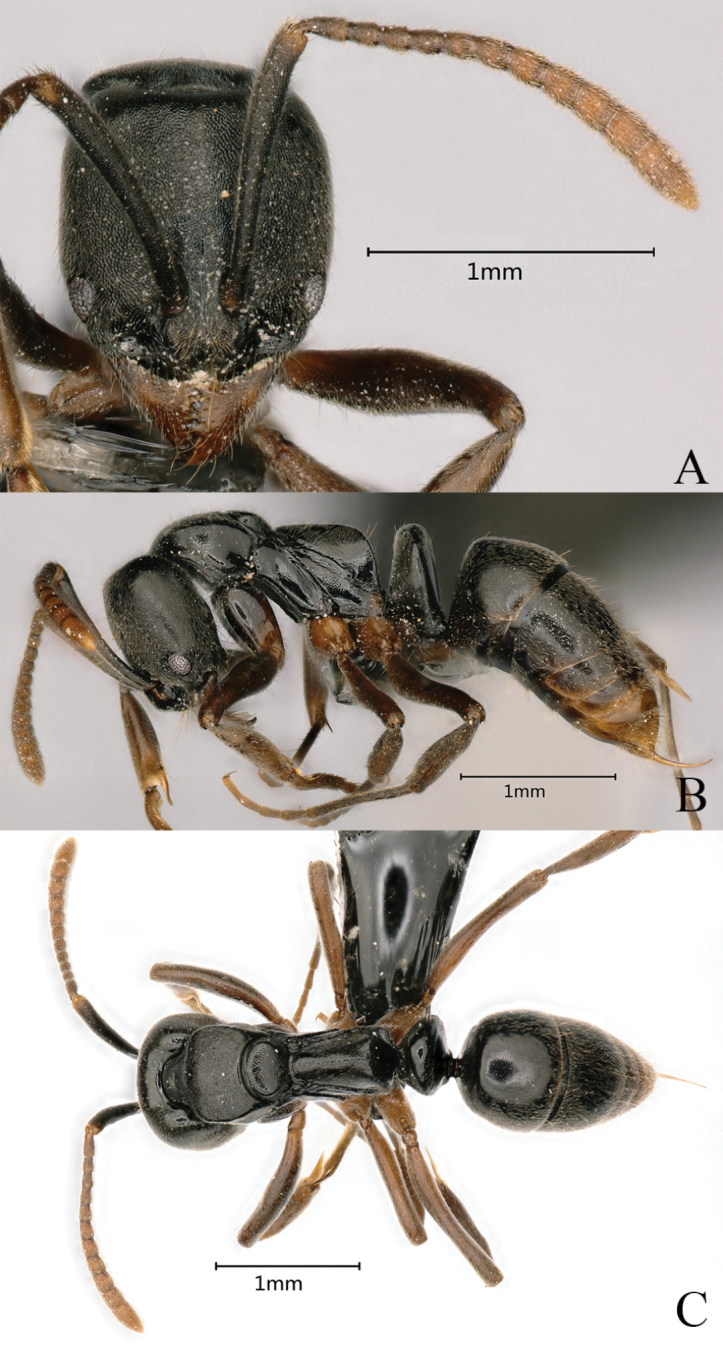
*Brachyponeraxui* sp. nov. worker (holotype, No. KIZ20231023) **A** head in full-face view **B** body in lateral view **C** body in dorsal view. Photographer Chao Chen.

***Holotype***: (Fig. 29A–C): HL 0.97, HLL 0.92, HLA 0.13, HW 0.96, ML 0.54, CML 0.08, CI 99, SL 0.97, SI 101, ED 0.16, PrL 0.65, PrH 0.48, PrW 0.67, WL 1.56, TL 4.9, PL 0.38, PH 0.78, DPW 0.56, LPI 205, PDPI 147. ***Paratypes*** (*n* = 2): HL 0.96–1.05, HLL 0.91–0.94, HLA 0.12–0.15, HW 0.96–1.04, ML 0.53–0.55, CML 0.08, CI 99–100, SL 0.97–1.01, SI 97–101, ED 0.14–0.17, PrL 0.63–0.67, PrH 0.45–0.50, PrW 0.66–0.69, WL 1.54–1.58, TL 4.8–5.1, PL 0.38–0.39, PH 0.77–0.79, DPW 0.55–0.58, LPI203–205, PDPI 145–148.

##### Diagnosis.

The new species is similar to *Brachyponerapilidorsalis* (Yamane), but it differs in head and pronotum with densely deeply punctate surface with small pits, eye relatively small, pronotum has edges (Fig. 9A), and dorsal surface of propodeum is longer. In *B.pilidorsalis*, head and pronotum are with sparsely lightly punctate sculpture with small pits, eye is relatively small, pronotum has weaker edges, and dorsal surface of propodeum is short.

##### Description.

In full-face view, head nearly square, with length and width roughly equal, posterior margin nearly straight, lateral margins moderately convex, posterolateral corners narrowly rounded. Mandible triangular with nine teeth, end tooth largest, and with a basal mandibular pit. Clypeus transverse, center of anterior margin moderately concave. Frontal carina short, frontal lobes well developed, covering antennal socket, frontal region with central longitudinal ridge. Antennae 12-segmented, scape exceeds posterolateral corner of head by 1/5 of its length, flagellar segments gradually increase in size toward the end. Eye medium and maximum diameter consists of nine ommatidia (ED 0.16 mm).

In lateral view, lateral edge of pronotum has obvious edges. Pronotum and mesonotum significantly higher than propodeum. Promesonotal suture seams evident, metanotal groove deeply impressed. Mesonotum moderately convex. Dorsal surface of propodeum nearly straight, declivity steep slope, nearly straight, posterodorsal corner of propodeum broadly rounded. Propodeal spiracle rounded, there is a groove between spiracle and metanotal groove. Metapleural bulla small, roughly elliptical. Petiolar node as high as propodeum, upright, nearly trapezoidal; anterior margin nearly straight, posterior margin and dorsum moderately convex; subpetiolar process forms a wedge. Prora absent. Gaster subconical, basal two intersegments contracted, apex with sting.

In dorsal view, lateral edge of pronotum has obvious edges; equal width between upper and lower parts; pronotum broadest, lateral margins gradually narrower posteriorly. Mesonotum anterior margin convex, posterior margin slightly concave. propodeum nearly rectangular, gradually narrowing from the bottom to the top, forming a ridge. Petiolar node broader than long, anterior margin moderately convex, lateral margins strongly convex, posterior margin flat.

Mandible with sparsely punctate with small pits, head and pronotum with densely punctate with small pits. lateral face of pronotum, propodeum, petiolar node and gaster with hairy punctation. Mesopleuron, metapleural and lower part of lateral side of petiolar node smooth and shiny. dorsal surface of body with sparsely erect or suberect hairs and densely sub-decumbent hairs. Body color black, funiculus, mandible, and legs yellowish brown.

##### Ecological notes.

The new species was collected in the Huanglianshan National Nature Reserve in Yunnan. The type series was collected while foraging on the ground surface of a montane mossy evergreen broadleaf forest at an altitude of 1750 m (Suppl. material 1: fig. S19), and one paratype was collected while foraging under a rock. One paratype was collected while foraging on the surface of a monsoon evergreen broad-leaved forest at an elevation of 750 m (Suppl. material 1: fig. S20). Another 107 workers of this new species were collected in seven sample plots in forest types including tropical seasonal rainforest, monsoon evergreen broadleaf forest, montane mossy evergreen broadleaf forest, mixed coniferous broadleaf forest, and deciduous broadleaf forest. Nesting sites included under rocks, in rotten wood, under rotten wood, and in soil. Foraging sites included surface and under stones. All sample plots were below 2000 m in elevation.

##### Etymology.

The new species is named in honor of Professor Zhenghui Xu (Southwest Forestry University, China) for his outstanding contributions to the ant fauna of China.

### ﻿Comments on the taxonomic position of *Euponeratianzun* (Terayama, 2009), comb. nov.

#### ﻿ Euponera tianzun

Taxon classificationAnimaliaHymenopteraFormicidae

(Terayama, 2009) comb. nov.

04457C84-E4AE-596B-AAEC-0B97F47780F7

Fig. 30A–D

Pachycondyla tianzun Terayama, 2009: 106, figs 31–35, 38 (w.). Type locality: Taiwan.
Brachyponera tianzun (Terayama, 2009): Schmidt and Shattuck 2014: 81.

##### Type material.

Holotype worker of *E.tianzun* was examined in online database of the NIAES (http://www.niaes.affrc.go.jp/inventory/insect/inssys/hymlst.htm HYM-182, imaged by Hiraku Yoshitake & Takashi Kurihara). ***Holotype*** (worker). Type locality: China: Taiwan Province, Nantou Pref., Nanfeng-Cun, Nanshanxi, 12. viii. 1980 (M. Terayama). Type-depository: NIAES (National Institute for Agro- Environmental Sciences).

##### Diagnosis.

This species is separated from *Euponerasharpi* Forel, 1901 by the angulate posterodorsal corner and much steeply sloped posterior margin of propodeum. Rather it resembles *E.sakishimensis* (Terayama, 1999) from the Ryukyus, Japan, and *E.pilosior* (Wheeler, 1928) from Japan and Korea. However, it is separated from the latter two by the narrow dorsal surface of propodeum and triangular subpetiolar process (Terayama 2009).

##### Measurements and indices.

***Holotype***: HL 1.18, HW 1.08, SL 0.80, WL 1.65, PL 0.40, PH 0.75, DPW 0.57, TL 4.7 CI 92, SI 74 (Terayama 2009).

##### Redescription of worker

**Figure 30.**
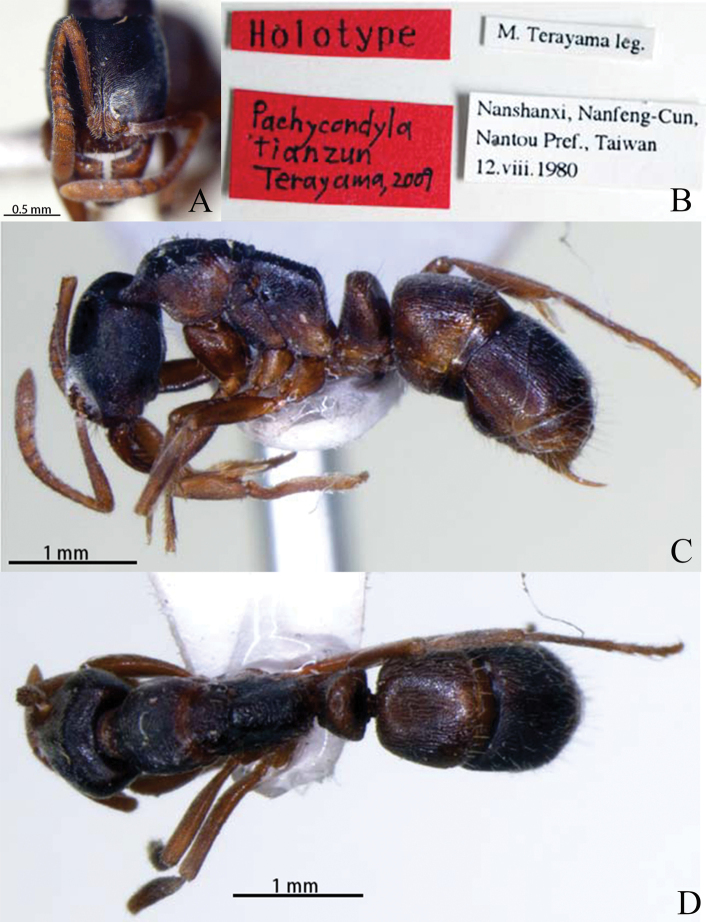
*Euponeratianzun* worker (holotype) **A** head in full-face view **B** labels **C** body in lateral view **D** body in dorsal view. Photographers Hiraku Yoshitake, Takashi Kurihara.

(Fig. 30A–D). In full-face view, head longer than broad, roughly rectangular, posterior margin weakly concave, posterolateral corners narrowly rounded, lateral margins moderately convex. Mandible triangular with ten teeth, and with a basal pit; masticatory margin meets with basal margin a right angle. Anterior margin of clypeus weakly convex, median clypeal ridge slightly raised. Frontal lobes well developed, covering antennal socket. Antennae 12-segmented; scape slightly exceeding posterior margin of head; flagellum obviously incrassate toward apex. Eye small, consisting of six or seven ommatidia, 0.04 mm in diameter.

In lateral view, dorsal and ventral margins of head slightly convex. Promesonotal suture and metanotal groove impressed. Mesonotum slightly convex. Dorsal surface of propodeum nearly straight, posterodorsal corner narrowly rounded, declivity steeply sloping (almost vertical), about equal to length of dorsum. Spiracle circular and located at about midpoint of propodeal side. Metapleural bulla roughly elliptical. Petiolar node higher than long; in dorsal view anterior margin nearly straight and dorsal and posterior margins slightly convex; subpetiolar process triangular, with dully angulate ventral corner. Prora present on anterior margin of first gastral sternite. Gaster roughly elliptical, apex with sting.

With mesosoma in dorsal view, pronotum broadest, lateral margin moderately convex, humeral corner indistinct. Promesonotal suture and metanotal groove impressed. Mesonotum elliptical, its lateral margin moderately convex. Petiolar node broader than long, anterior and lateral margins slightly convex, posterior margin nearly straight.

Head and body surface weakly punctate. Clypeus, pronotum, petiolar node and gaster with sparse erect hairs, cranium, antennae, mesosoma and gaster with suberect hairs and pubescence. Body dark reddish brown to blackish brown; head darker than alitrunk; antenna, mandible, and legs reddish brown.

##### Comments.

The species was transferred to *Euponera* because it bears morphological traits typical for this genus, among them: head rectangular, longer than wide (CI = 91), with concave posterior margin in full-face view; mandible with 10 teeth, and with a basal mandibular pit; anterior margin of clypeus weakly convex; antennal scape slightly exceeding the posterior margin of head; SI = 74; eye small, consisting of six or seven facets, 0.04 mm in diameter; alitrunk with straight dorsal margin in profile; propodeum with angulate posterodorsal corner and steeply sloped posterior margin in profile, in dorsal view dorsal surface relatively wide; petiole higher than long in profile, subpetiolar process triangular, with dully angulate ventral corner (Terayama 2009).

## ﻿Discussion

In this study, we described four new species of *Brachyponera* distributed in China, and we hypothesize that the number is not definitive as there are most likely still undiscovered species of occurring in this country. The distribution range of this genus covers Oriental, Indo-Australian, and Palaearctic regions (Janicki et al. 2016), and further studies should provide more insights into the diversity of *Brachyponera* on a global scale. Also, studies on this genus are challenging due to the small morphological differences between species. However, the four new species described in this work are distinguished based on strong and well-defined characters.

The species group division proposed by Yamane (2007) was supplemented with additional sets of characters that should facilitate taxonomic studies on this genus. In summary, we distinguish 23 species of *Brachyponera* divided into eight species groups, i.e., *atrata* group, *lutea* group, *kumtongi* group, *chinensis* group, *xui* group, *arcuata* group, *nigrita* group, and *obscurans* group. The complexity of biomes can be simplified by grouping species with the same characteristics into one species group, making it easier to study and understand the structure and function of ecosystems.

By sequencing, we obtained the DNA sequences of mitochondrial COI of four new species and *B.brevidorsa*. In addition, sequences of nine species, including two outgroups, were downloaded from GenBank. The analysis showed that the smallest genetic distance between species was 7% (*B.croceicornis* and *B.obscurans*), and the largest genetic distance was 19.62% (*B.nakasujii* and *B.paraarcuata* sp. nov.). However, our results should be viewed cautiously, as trees based only on COI are considered unreliable, often disagreeing with those inferred from ultraconservative genes (Hebert et al. 2003; Pons et al. 2006). Collecting more specimens in subsequent surveys will allow for building a more complete phylogenetic tree and the future analyses should be based on UCEs. By this, it will be more likely to gain a better understanding of the phylogeny of *Brachyponera*.

During the field survey, we did not collect enough queens and males, which resulted in our inability to understand the complete life history of each species fully. We believe that further research on the *Brachyponera* will provide new insights into the morphological classification, genetic evolution, and biology of the genus.

## Supplementary Material

XML Treatment for Brachyponera brevidorsa

XML Treatment for Brachyponera candida

XML Treatment for Brachyponera chinensis

XML Treatment for Brachyponera luteipes

XML Treatment for Brachyponera myops

XML Treatment for Brachyponera nigrita

XML Treatment for Brachyponera obscurans

XML Treatment for Brachyponera paraarcuata

XML Treatment for Brachyponera xui

XML Treatment for Euponera tianzun

## ﻿Additional information

### Author ORCIDs

Chao Chen https://orcid.org/0009-0007-2983-9570

Yu Yu https://orcid.org/0009-0002-8775-1415

Chuanhui Yi https://orcid.org/0009-0008-9014-9947

### Data availability

All of the data that support the findings of this study are available in the main text or Supplementary Information.

## Supplementary materials

Supplementary material 1 Additional images Data type pdf https://binary.pensoft.net/file/1271856This dataset is made available under the Open Database License (http://opendatacommons.org/licenses/odbl/1.0/). The Open Database License (ODbL) is a license agreement intended to allow users to freely share, modify, and use this Dataset while maintaining this same freedom for others, provided that the original source and author(s) are credited.Chao Chen, Yu Yu, Chuanhui Yi
