# Supplementary material for: ﻿Revision of the Chinese species of the genus Brachyponera Emery, 1900 (Hymenoptera, Formicidae), with a key to the world species of the genus
Source: Zookeys. 2025 Mar 6;1230:247–86. doi: 10.3897/zookeys.1230.140159 (PMC11907243; doi:10.3897/zookeys.1230.140159)
Supplement: Supplementary material 1 — Additional images [file zookeys-1230-247_article-140159__-s001.pdf]

Supplementary figures

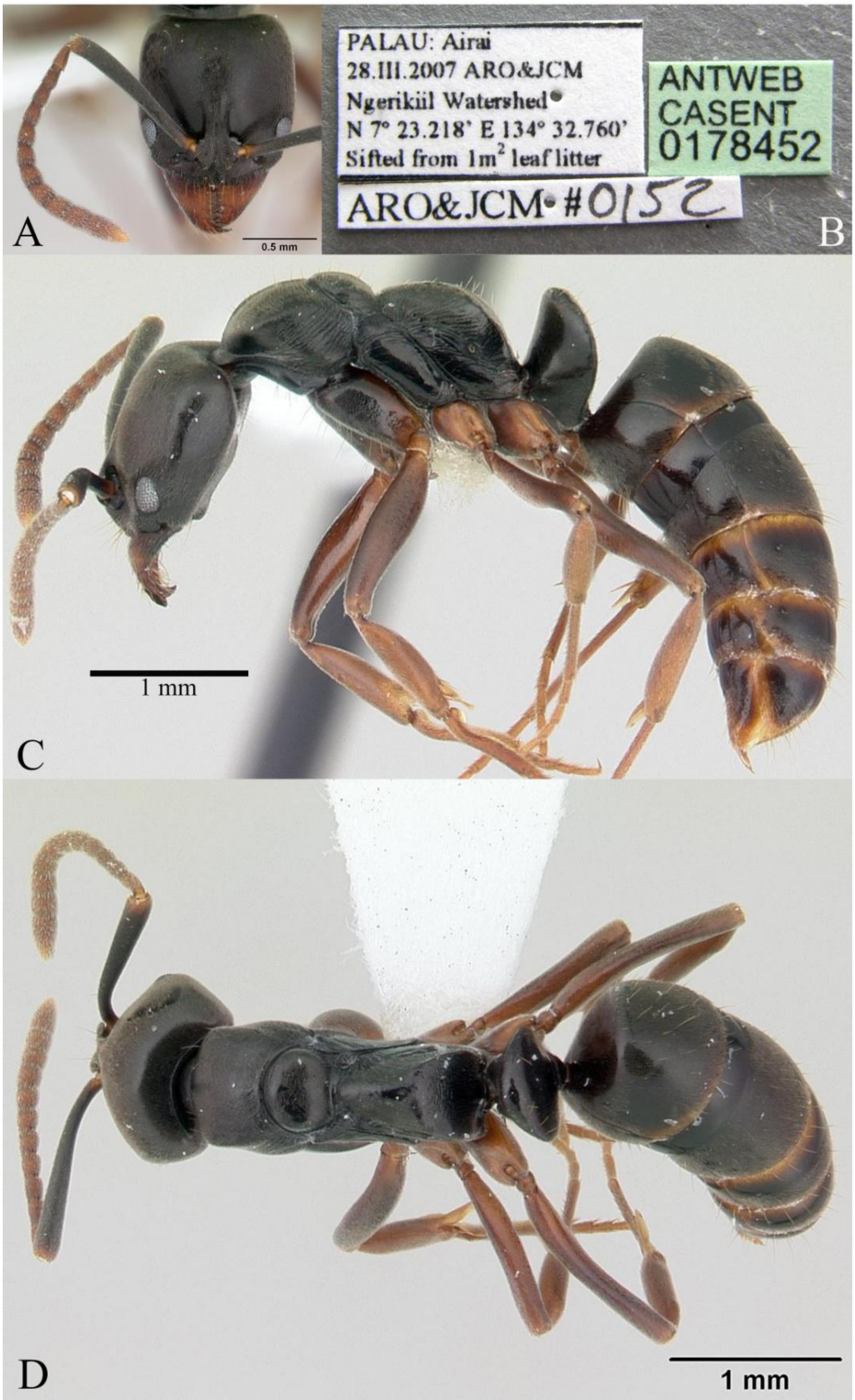

Figure S1. *Brachyponera atrata* worker (Non-type, images cited from https://www.antweb.org/, CASENT0178452, imaged by April Nobile) (A) head in full-face view (B) label (C) body in lateral view (D) body in dorsal view .

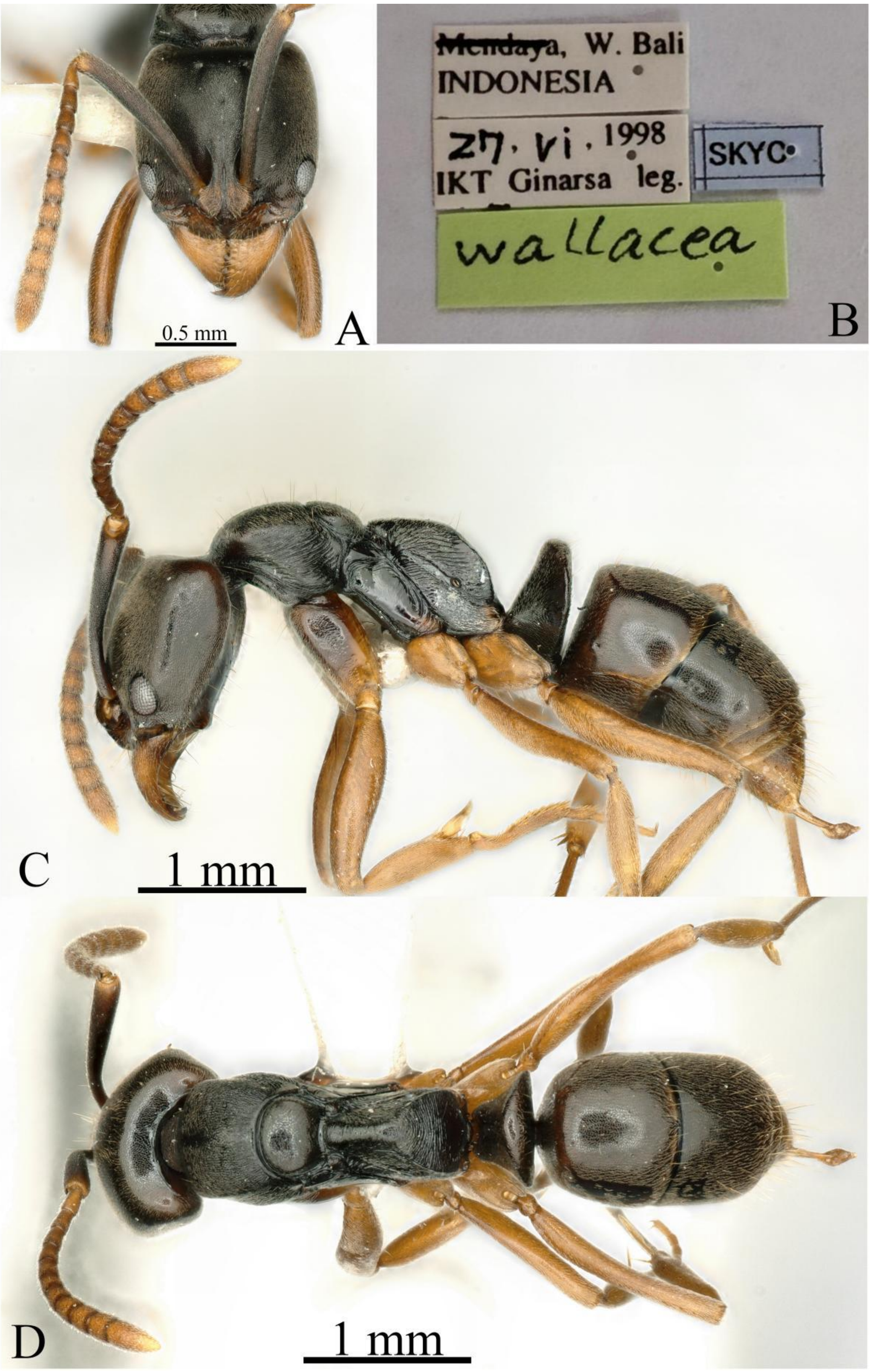

Figure S2. *Brachyponera wallacea* worker (Non-type, imaged by Chao Chen) (A) head in full-face view (B)label (C) body in lateral view (D) body in dorsal view

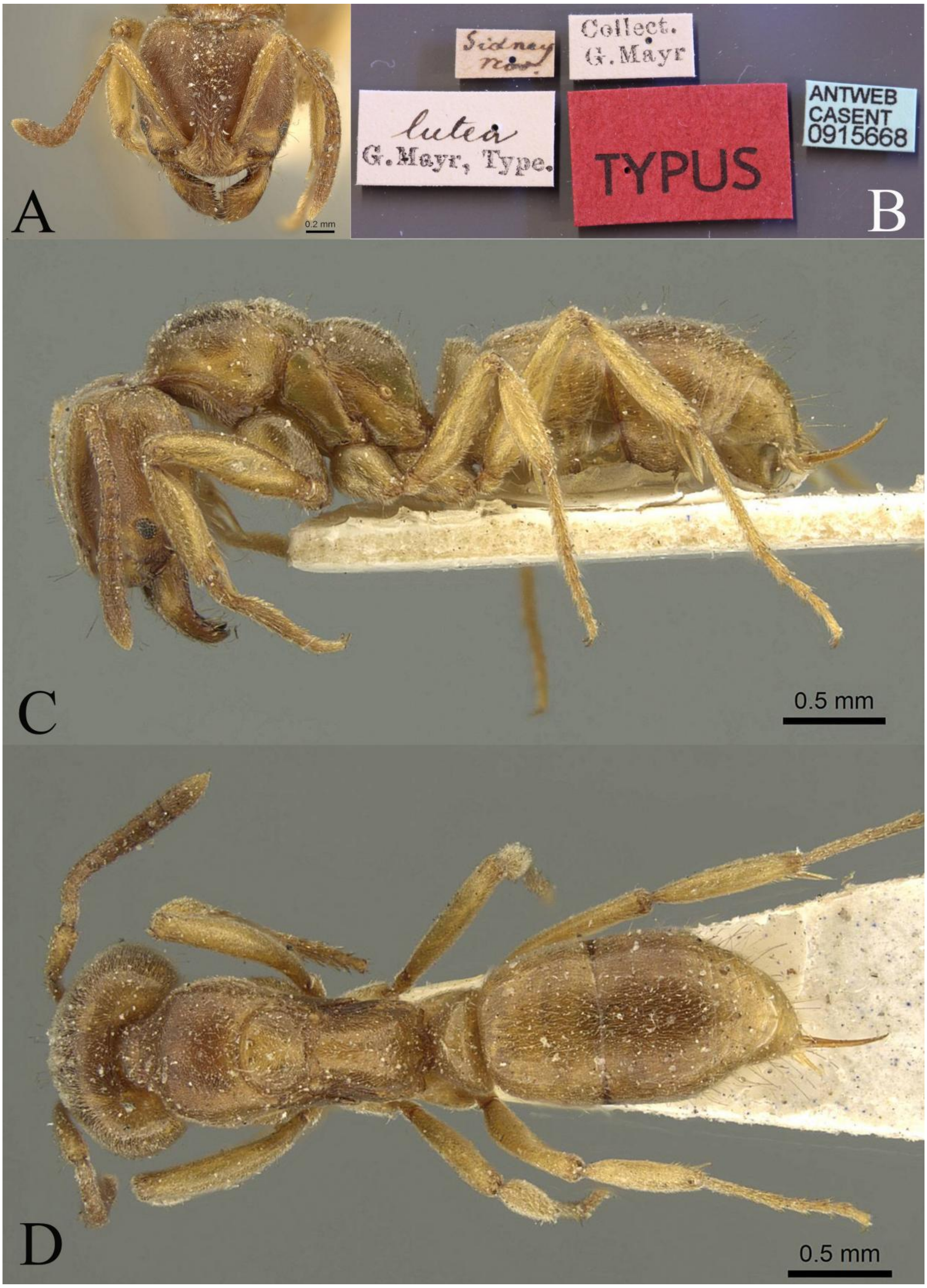

Figure S3. *Brachyponera lutea* worker (Type, images cited from https://www.antweb.org/, CASENT0915668, imaged by Harald Bruckner) (A) head in full-face view (B) label (C) body in lateral view (D) body in dorsal view .

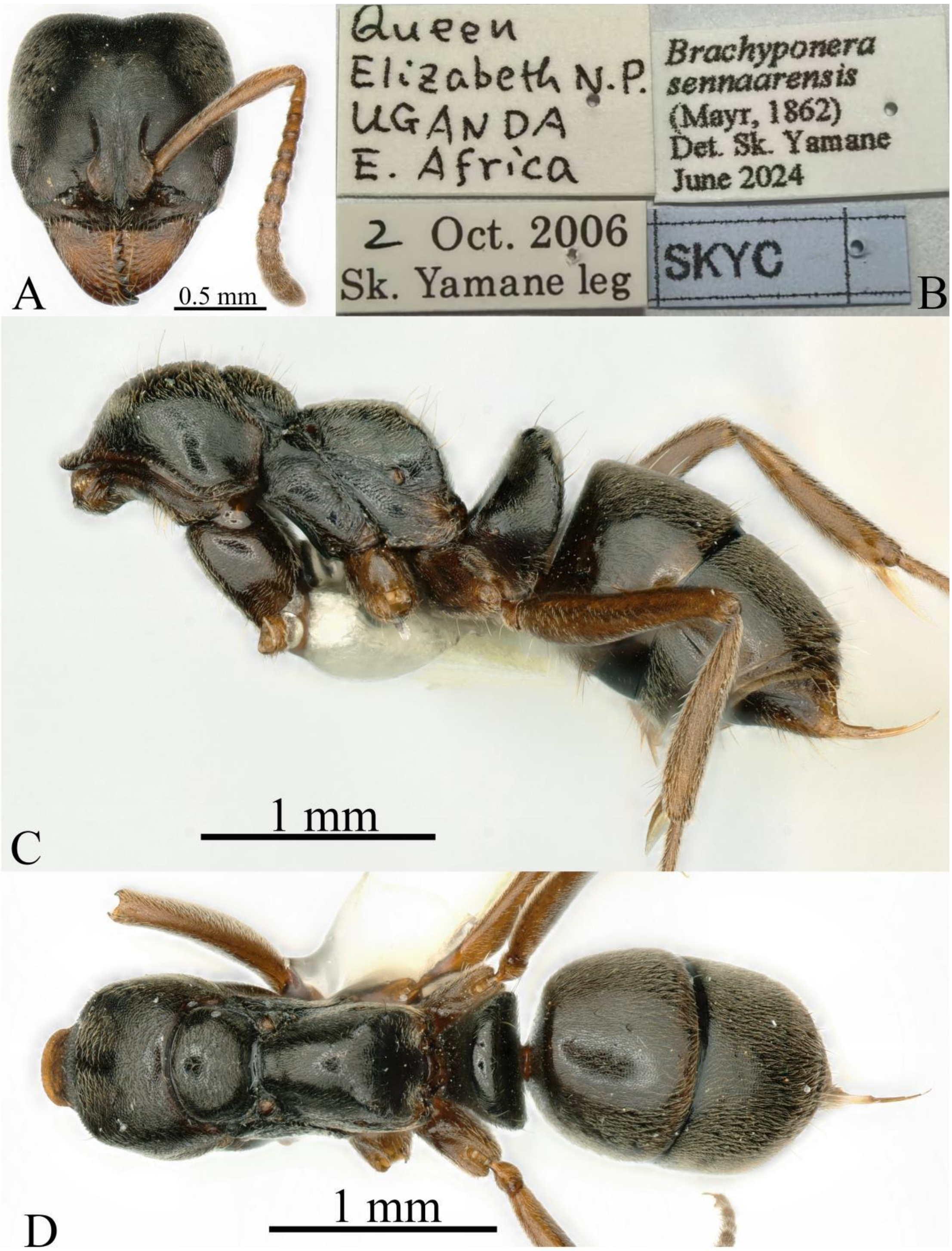

Figure S4. *Brachyponera sennaarensis* worker (Non-type, imaged by Chao Chen) (A) head in full-face view (B) label (C) body in lateral view (D) body in dorsal view

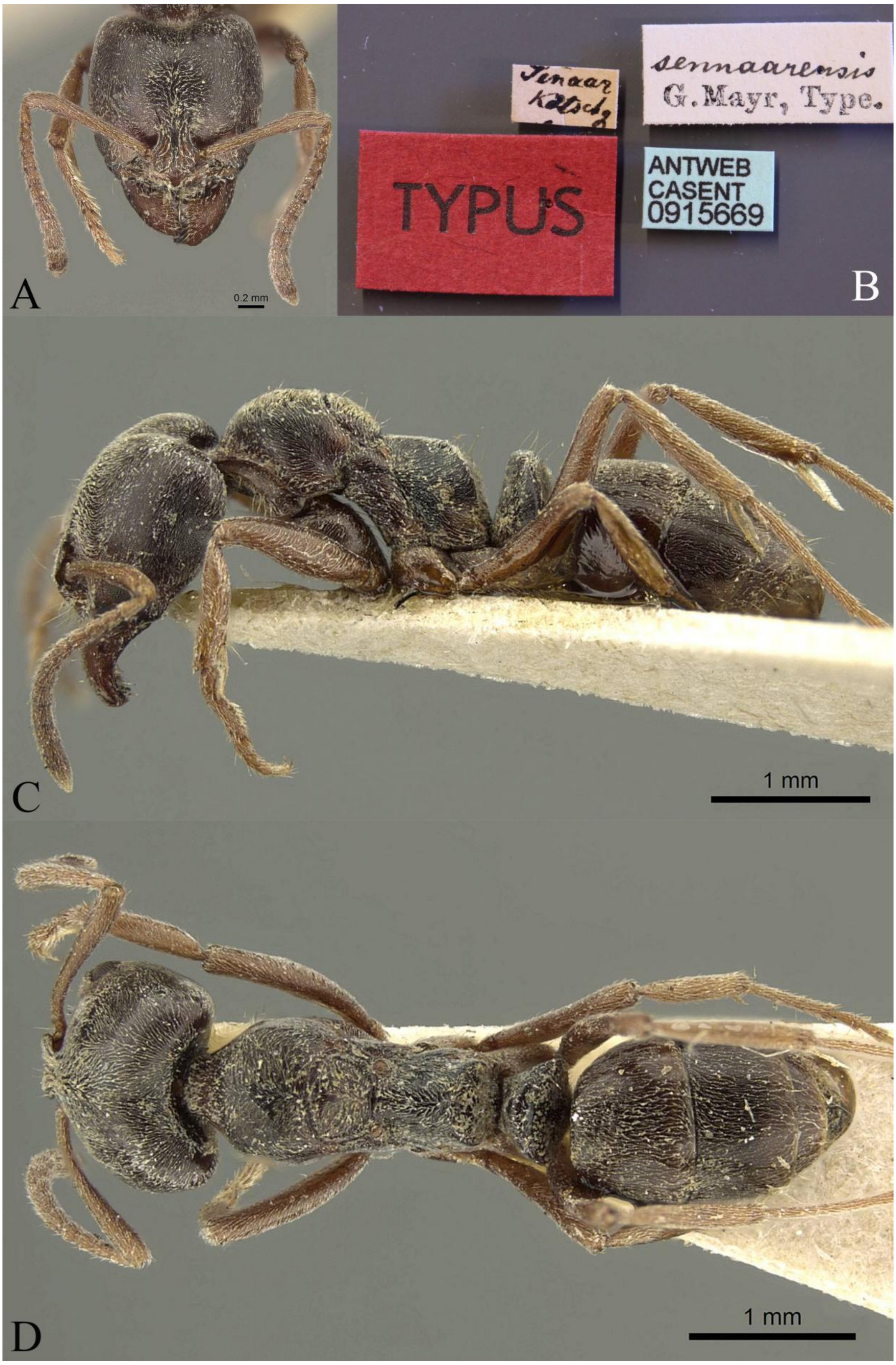

Figure S5. *Brachyponera sennaarensis* worker (Type, images cited from https://www.antweb.org/, CASENT0915669, imaged by Harald Bruckner) (A) head in full-face view (B) label (C) body in lateral view (D) body in dorsal view .

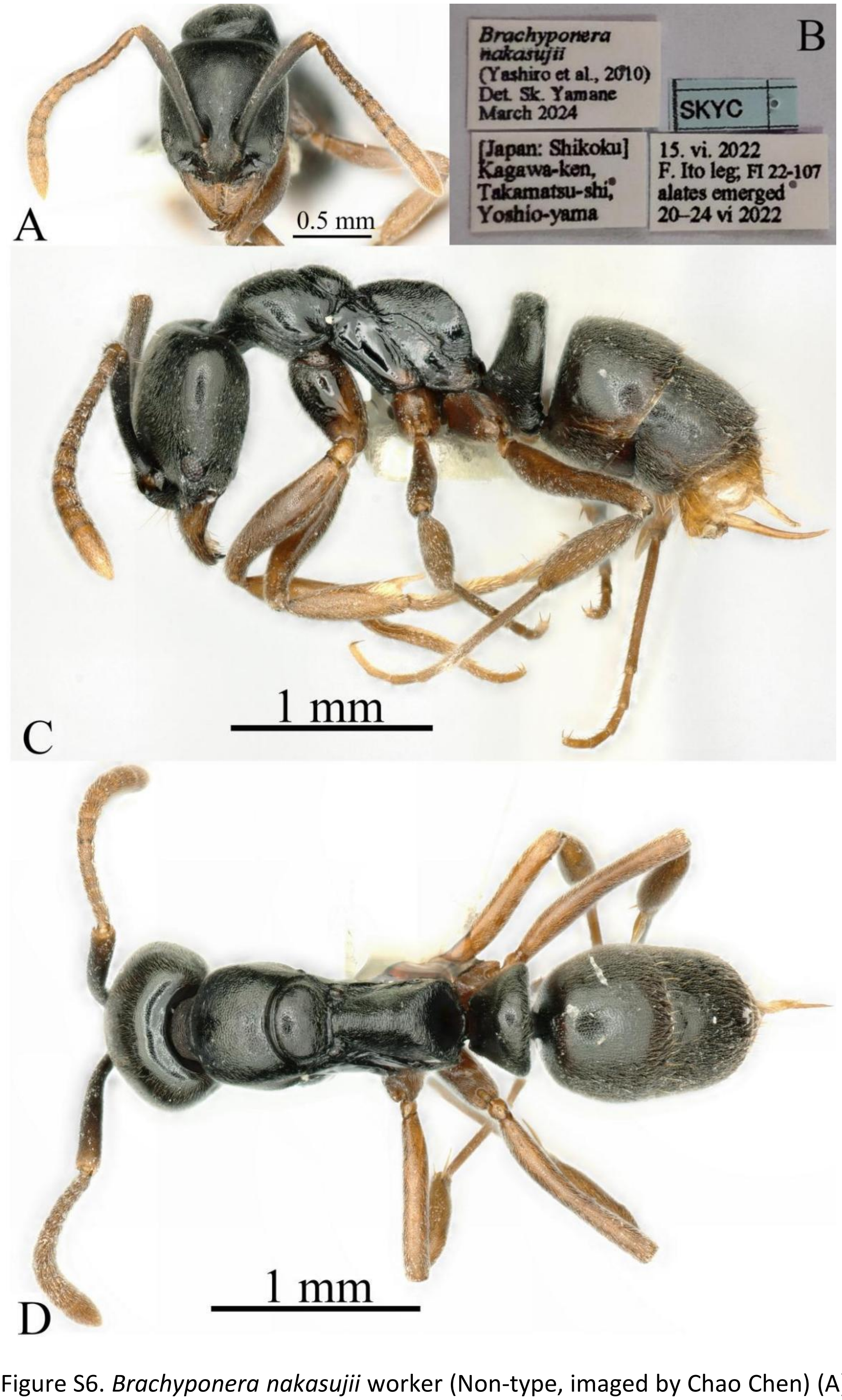

Figure S6. *Brachyponera nakasujii* worker (Non-type, imaged by Chao Chen) (A) head in full-face view (B) label (C) body in lateral view (D) body in dorsal view

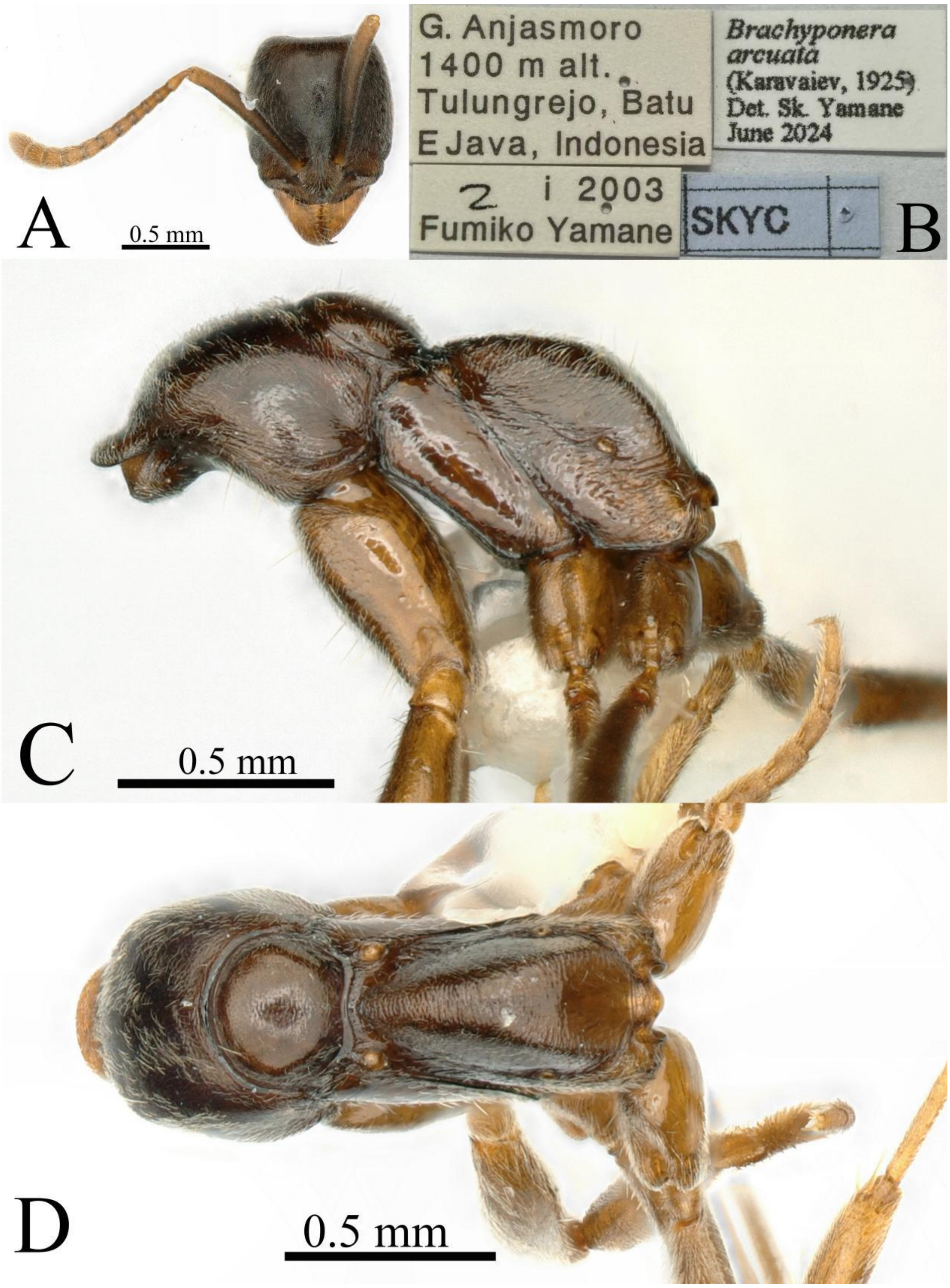

Figure S7. *Brachyponera arcuata* worker (Non-type, imaged by Chao Chen) (A) head in full-face view (B)label (C) body in lateral view (D) body in dorsal view

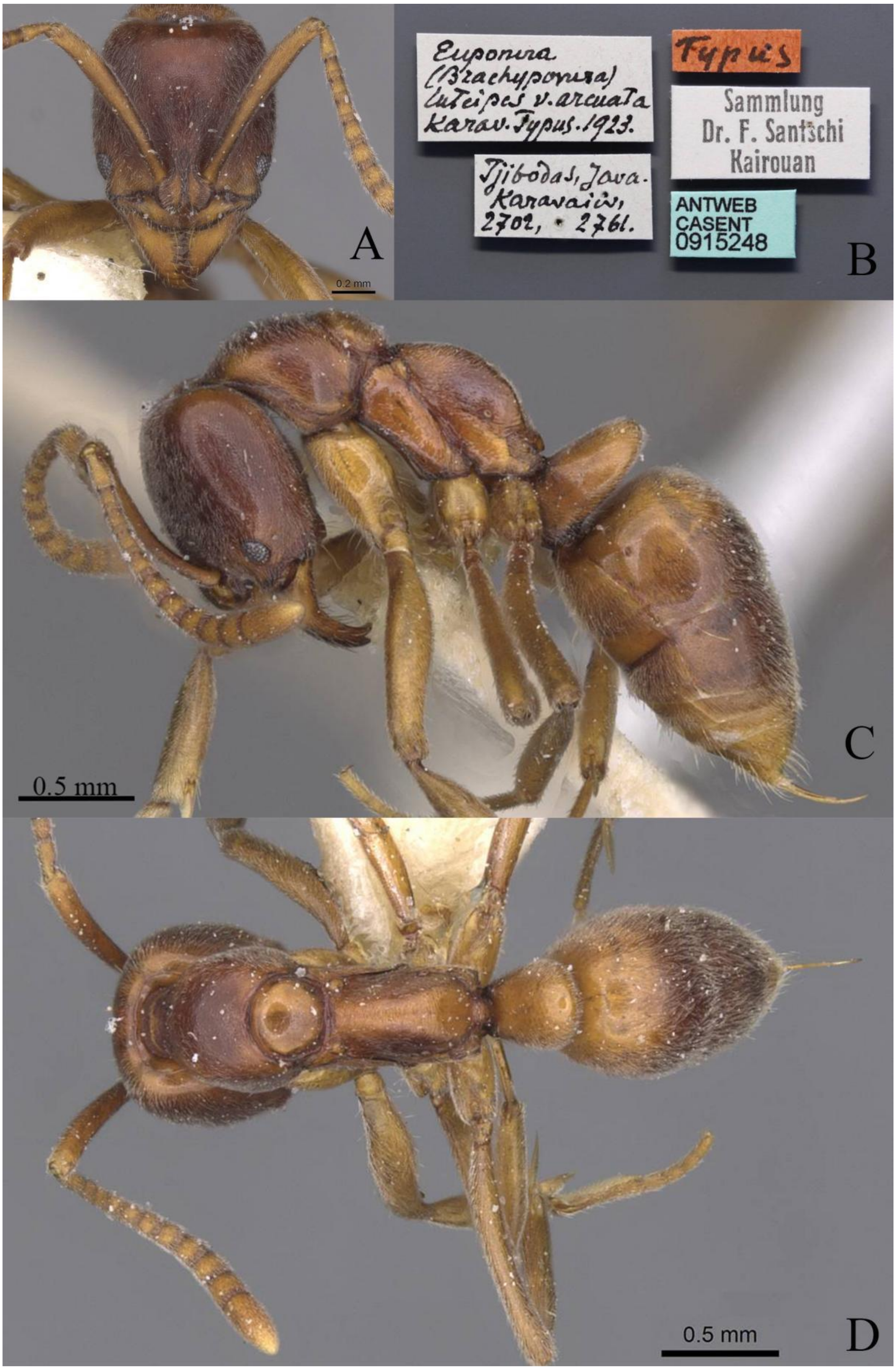

Figure S8. *Brachyponera arcuata* worker (Type, images cited from https://www.antweb.org/, CASENT0915248, imaged by Will Ericson) (A) head in full-face view (B) label (C) body in lateral view (D) body in dorsal view .

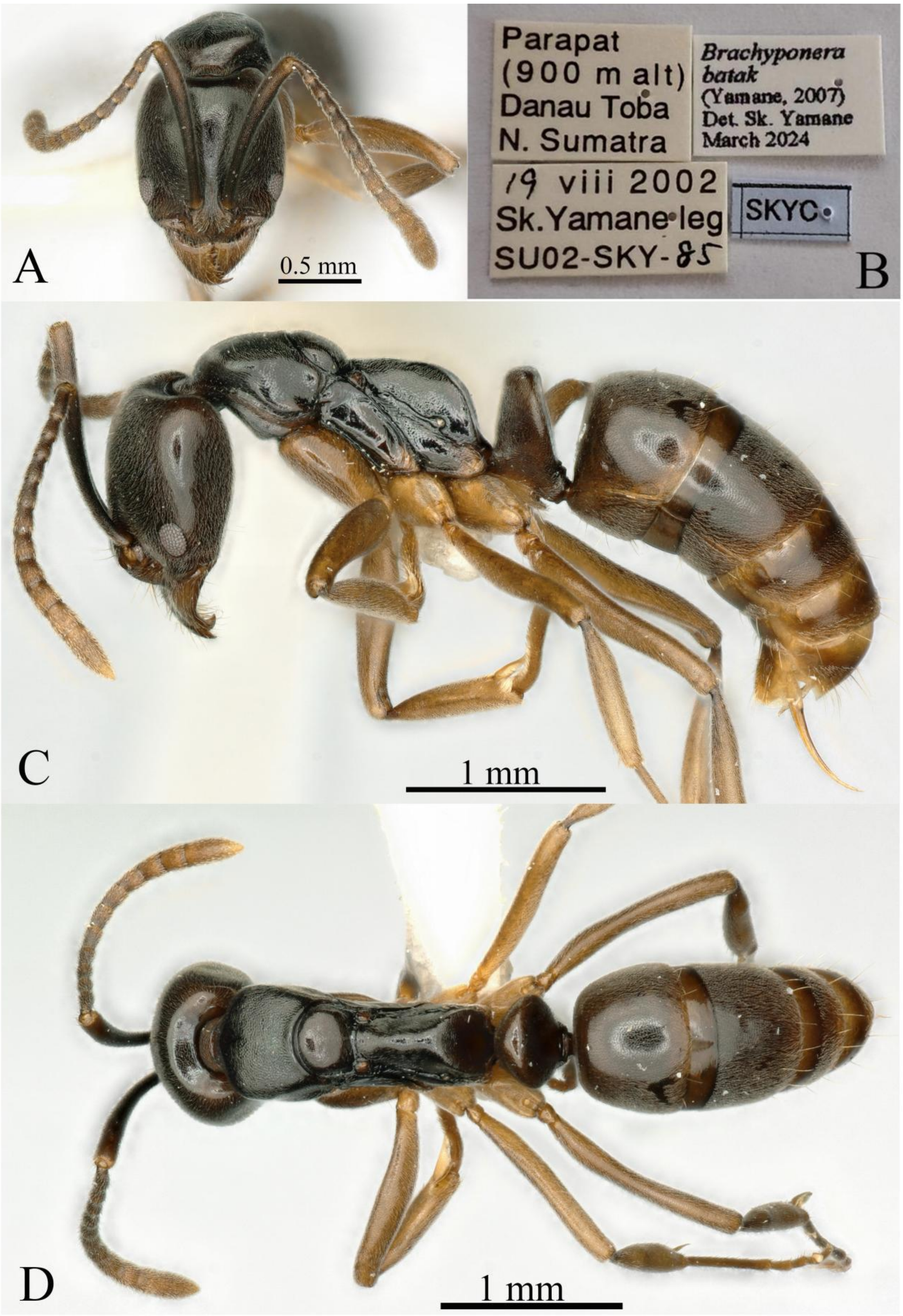

Figure S9. *Brachyponera batak* worker (Non-type, imaged by Chao Chen) (A) head in full-face view (B) label (C) body in lateral view (D) body in dorsal view

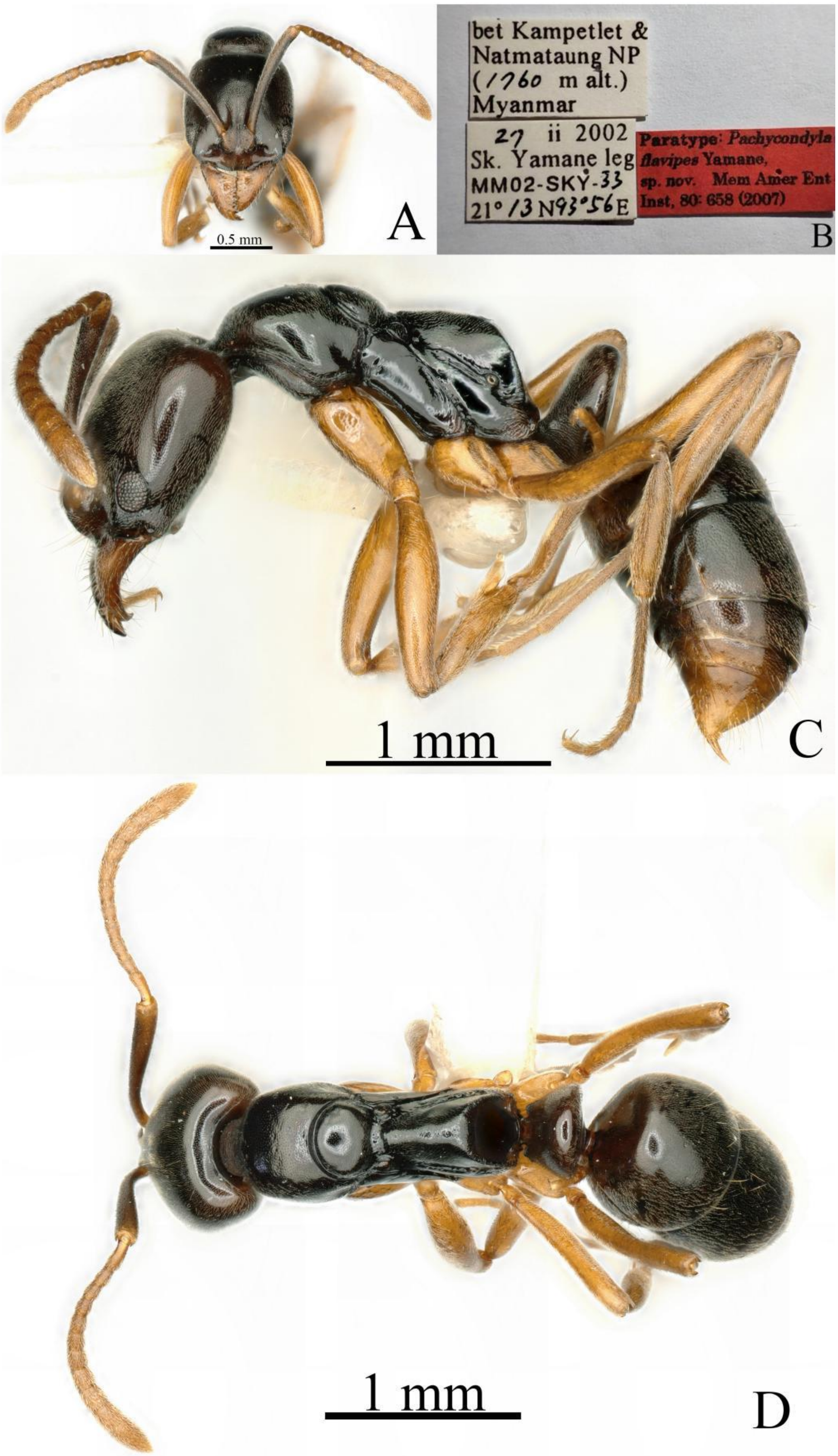

Figure S10. *Brachyponera flavipes* worker (Paratype, imaged by Chao Chen) (A) head in full-face view (B) label (C) body in lateral view (D) body in dorsal view

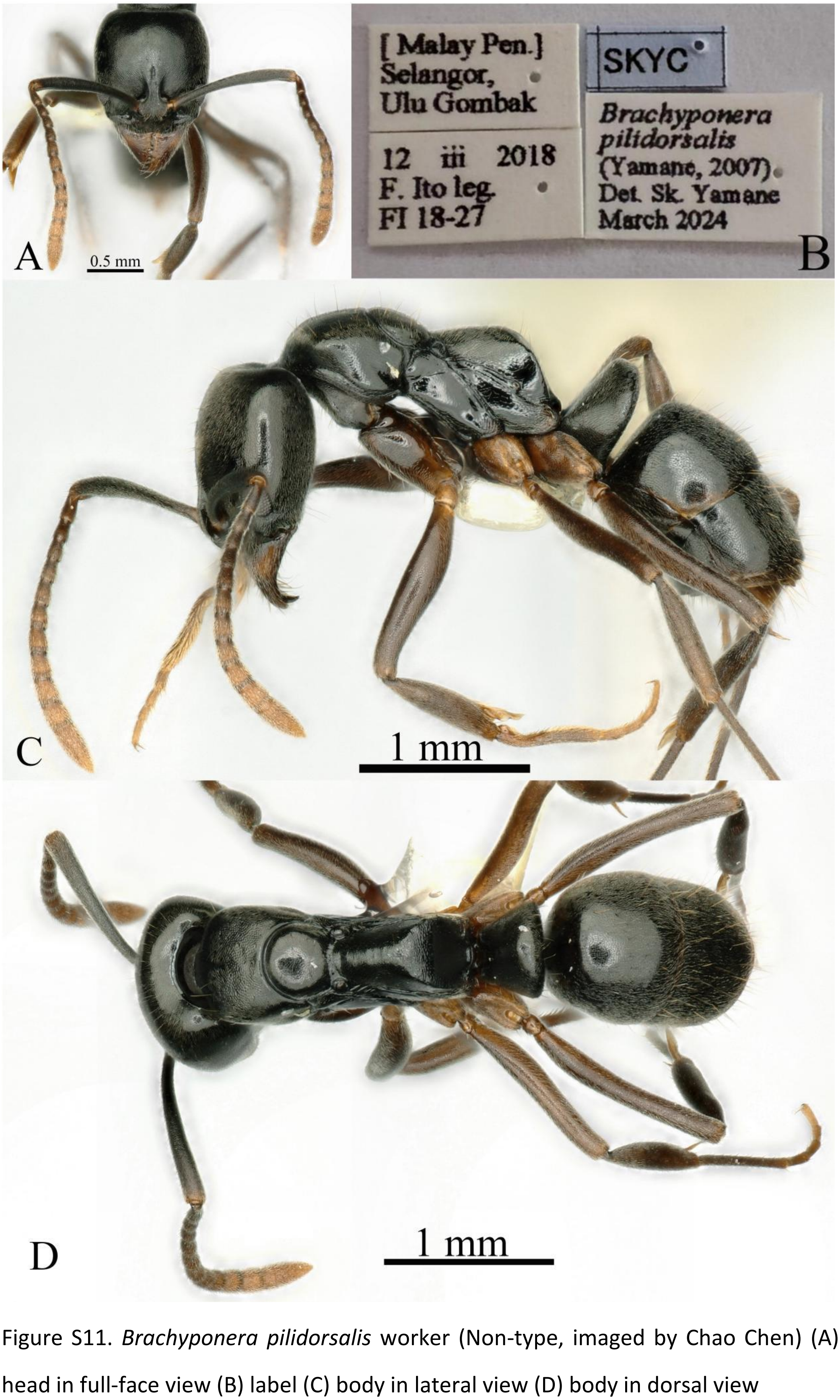

Figure S11. *Brachyponera pilidorsalis* worker (Non-type, imaged by Chao Chen) (A) head in full-face view (B) label (C) body in lateral view (D) body in dorsal view

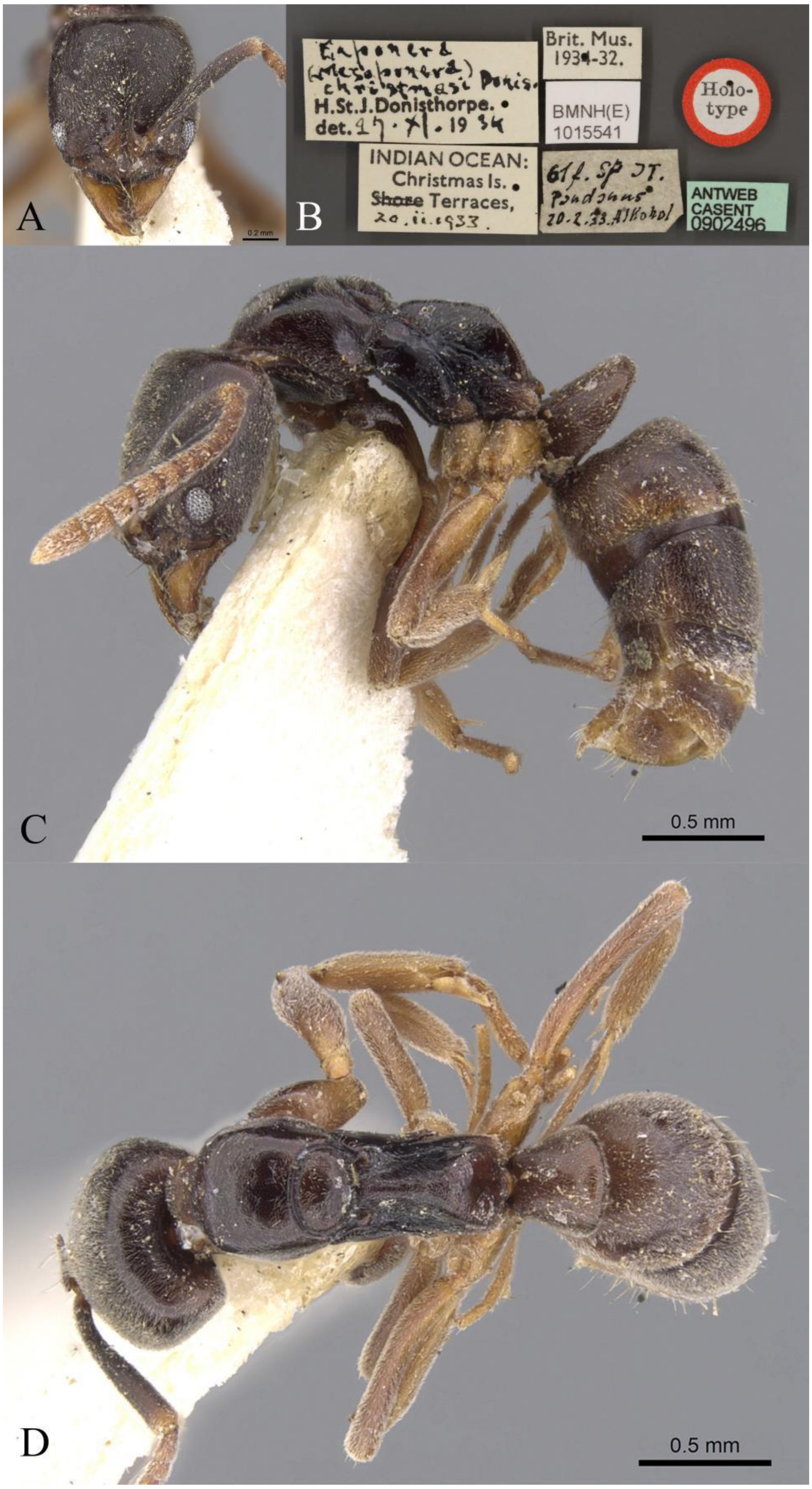

Figure S12. *Brachyponera christmasi* worker (Type, images cited from https://www.antweb.org/, CASENT0902496, imaged by Will Ericson) (A) head in full-face view (B) label (C) body in lateral view (D) body in dorsal view .

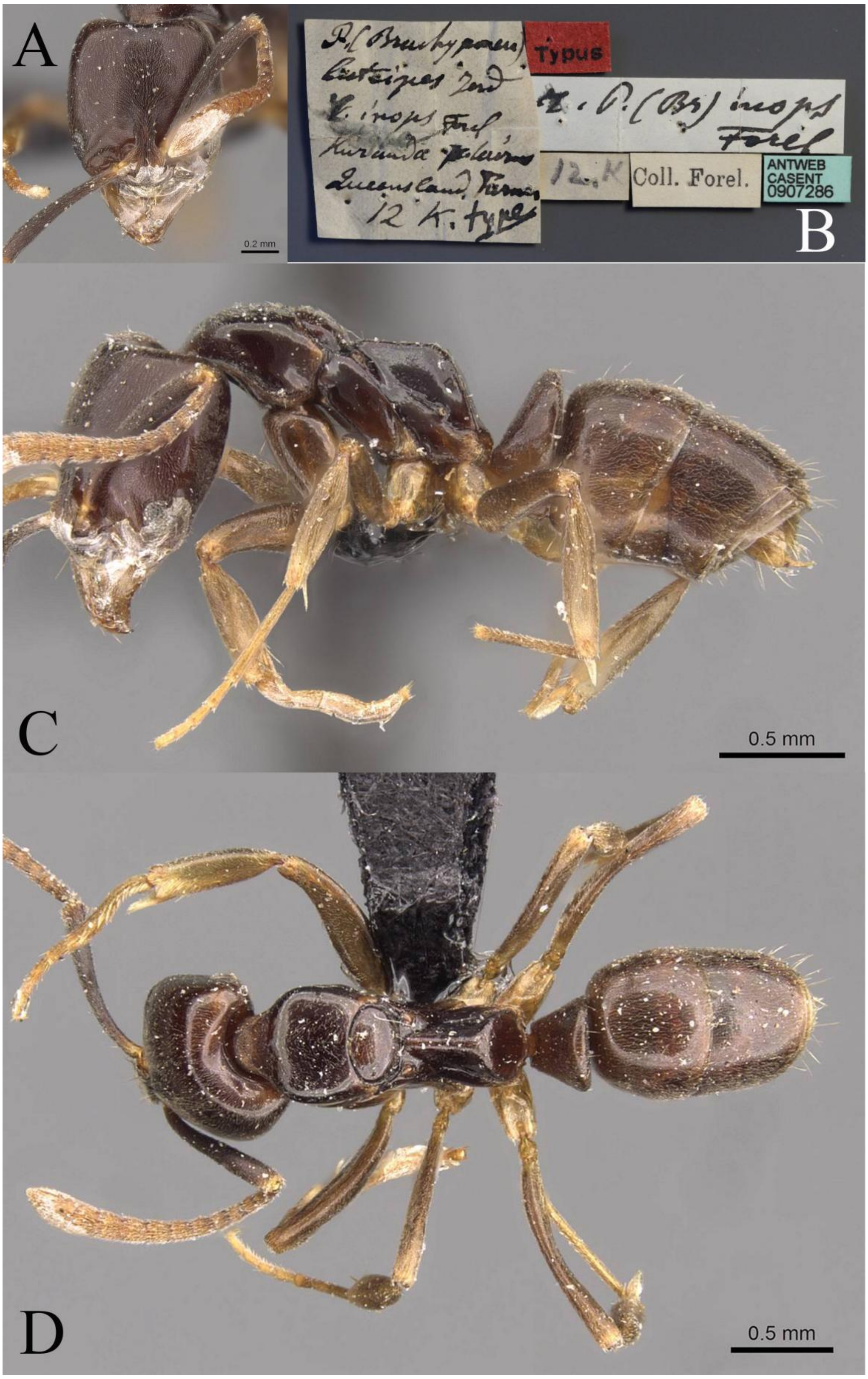

Figure S13. *Brachyponera croceicornis* worker (Type, images cited from https://www.antweb.org/, CASENT0907286, imaged by Z. Lieberman) (A) head in full-face view (B) label (C) body in lateral view (D) body in dorsal view .

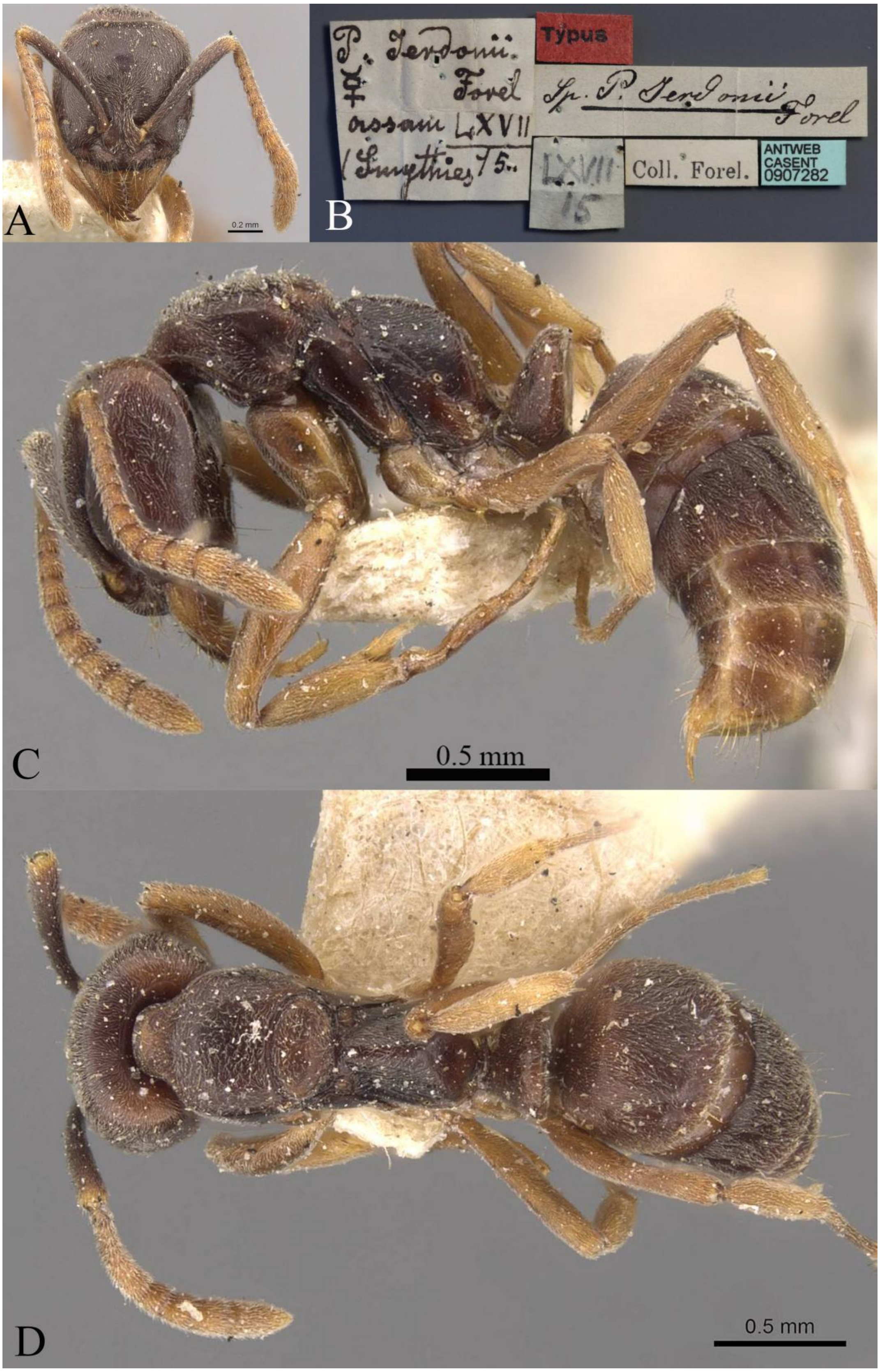

Figure S14. *Brachyponera jerdonii* worker (Type, images cited from https://www.antweb.org/, CASENT0907282, imaged by Z. Lieberman) (A) head in full-face view (B) label (C) body in lateral view (D) body in dorsal view .

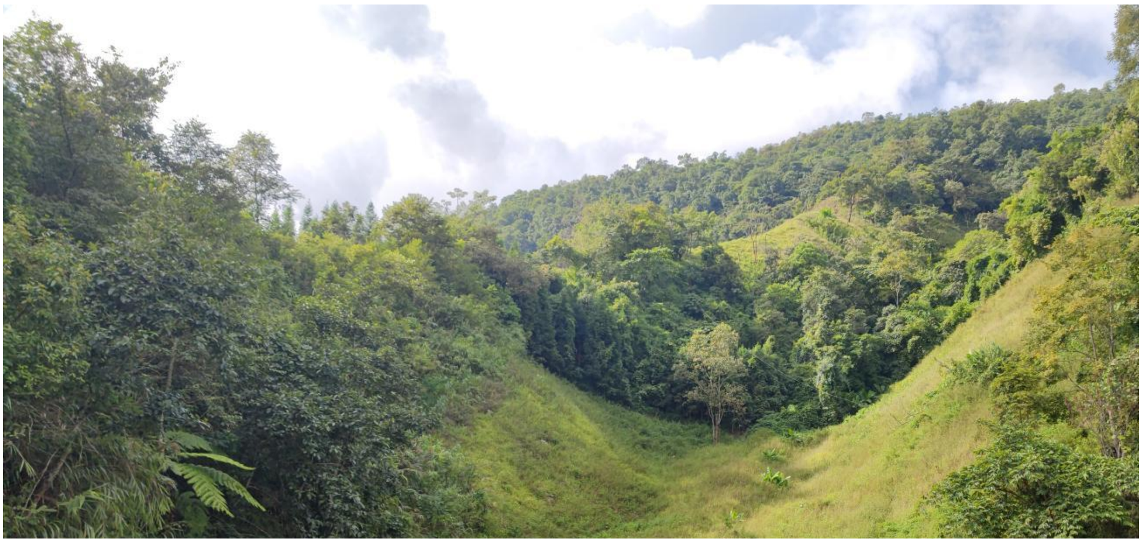

Figure S15. Habitat at the type locality of *Brachyponera candida* **sp. nov.**

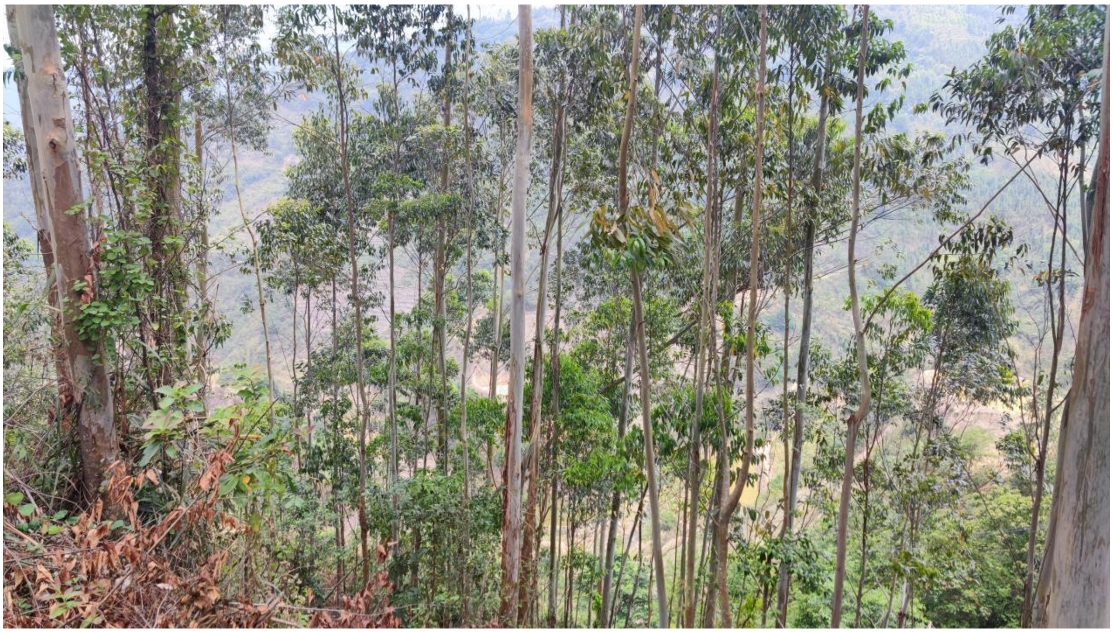

Figure S16. Habitat at the type locality of *Brachyponera myops* **sp. nov.**

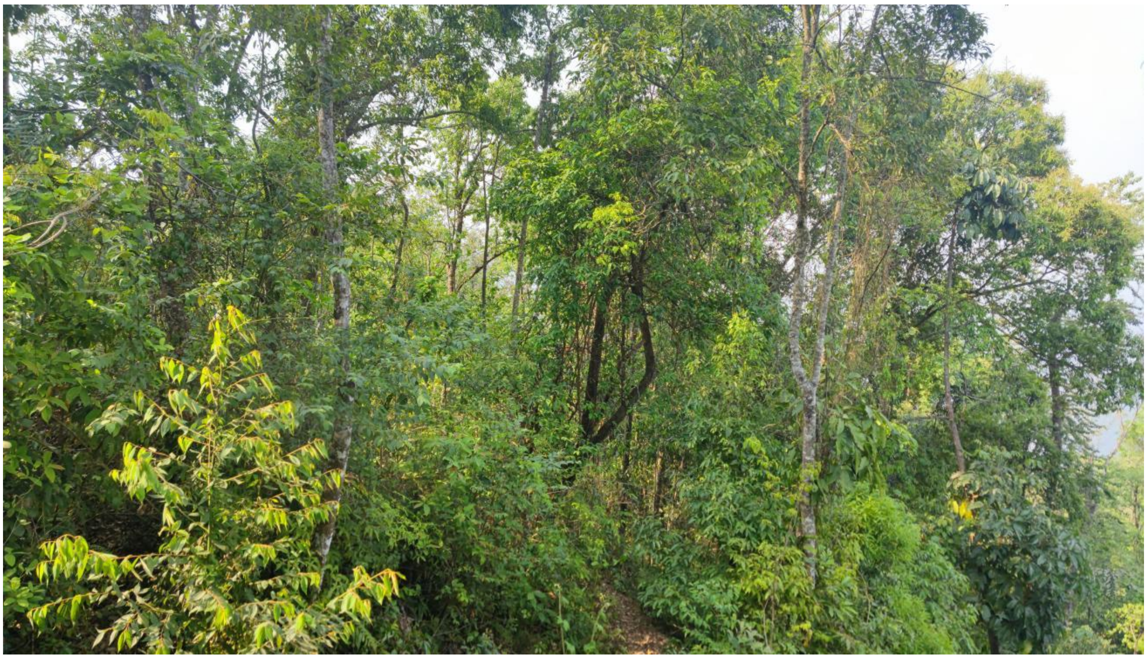

Figure S17. Habitat at the type locality of *Brachyponera myops* **sp. nov.**

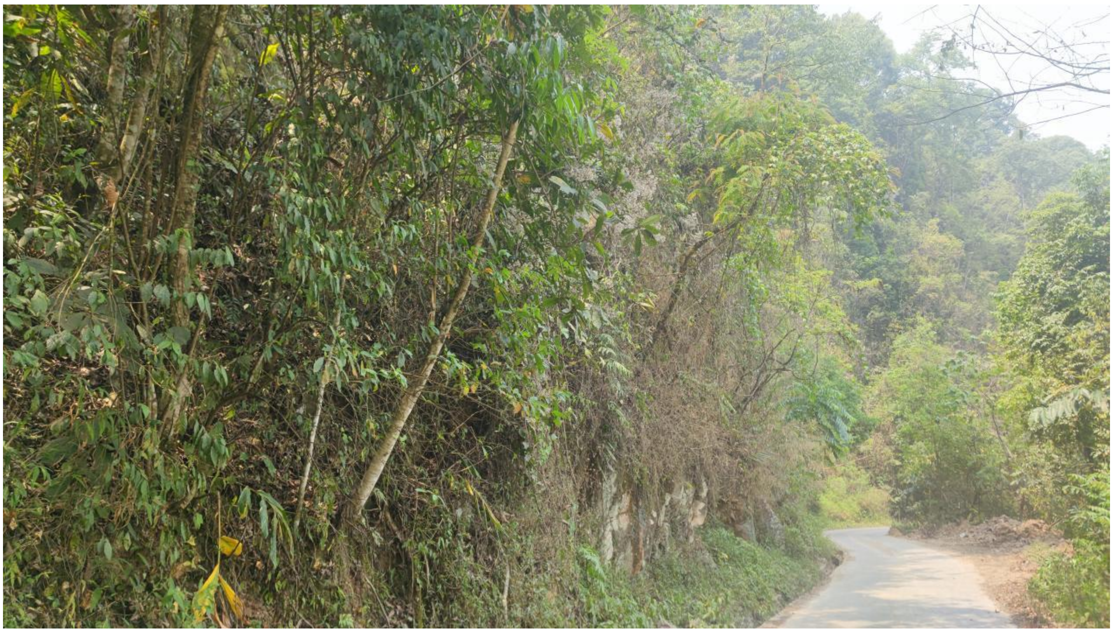

Figure S18. Habitat at the type locality of *Brachyponera paraarcuata* **sp. nov.**

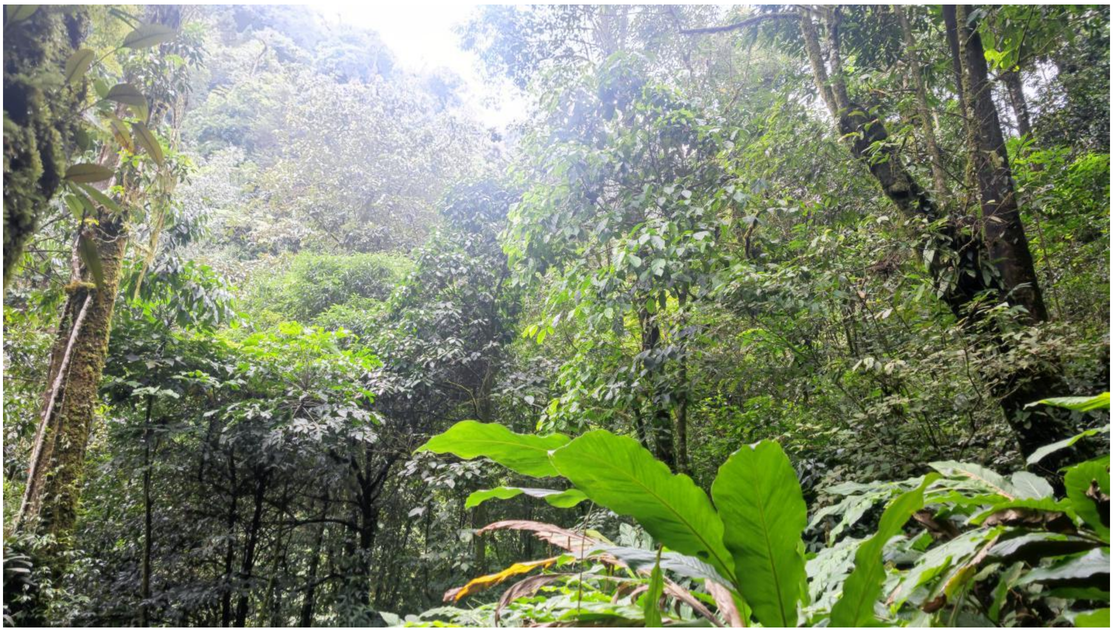

Figure S19. Habitat at the type locality of *Brachyponera xui* **sp. nov.**

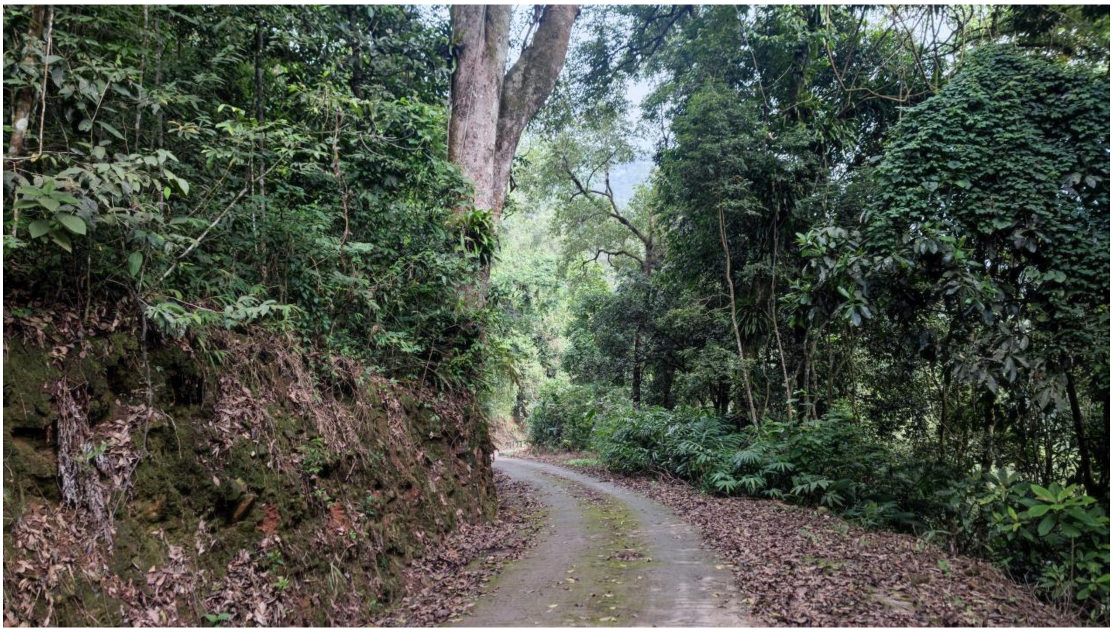

Figure S20. Habitat at the type locality of *Brachyponera xui* **sp. nov.**
